# Mice harboring the FXN I151F pathological point mutation present decreased frataxin levels, a Friedreich ataxia-like phenotype, and mitochondrial alterations

**DOI:** 10.1007/s00018-021-04100-5

**Published:** 2022-01-17

**Authors:** Marta Medina-Carbonero, Arabela Sanz-Alcázar, Elena Britti, Fabien Delaspre, Elisa Cabiscol, Joaquim Ros, Jordi Tamarit

**Affiliations:** Dept. Ciències Mèdiques Bàsiques, Fac. Medicina, IRBLleida, Universitat de Lleida, Av. Rovira Roure, 80, 25198 Lleida, Spain

**Keywords:** Friedreich Ataxia, Iron–sulfur, Mitochondria, Oxidative stress, OXPHOS

## Abstract

Friedreich Ataxia (FA) is a rare neuro-cardiodegenerative disease caused by mutations in the frataxin (*FXN*) gene. The most prevalent mutation is a GAA expansion in the first intron of the gene causing decreased frataxin expression. Some patients present the GAA expansion in one allele and a missense mutation in the other allele. One of these mutations, FXNI154F, was reported to result in decreased content of mature frataxin and increased presence of an insoluble intermediate proteoform in cellular models. By introducing this mutation into the murine *Fxn* gene (I151F, equivalent to human I154F) we have now analyzed the consequences of this pathological point mutation in vivo. We have observed that FXN^I151F^ homozygous mice present low frataxin levels in all tissues, with no evidence of insoluble proteoforms. Moreover, they display neurological deficits resembling those observed in FA patients. Biochemical analysis of heart, cerebrum and cerebellum have revealed decreased content of components from OXPHOS complexes I and II, decreased aconitase activity, and alterations in antioxidant defenses. These mitochondrial alterations are more marked in the nervous system than in heart, precede the appearance of neurological symptoms, and are similar to those observed in other FA models. We conclude that the primary pathological mechanism underlying the I151F mutation is frataxin deficiency, like in patients carrying GAA expansions. Therefore, patients carrying the I154F mutation would benefit from frataxin replacement therapies. Furthermore, our results also show that the FXN^I151F^ mouse is an excellent tool for analyzing tissue-specific consequences of frataxin deficiency and for testing new therapies.

**Supplementary Information:**

The online version contains supplementary material available at 10.1007/s00018-021-04100-5.

## Introduction

Friedreich Ataxia (FA) is a rare, inherited recessive disease first described by Nikolaus Friedreich, a German pathologist, in 1863. FA is characterized by progressive gait and limb ataxia with associated limb muscle weakness, absent lower limb reflexes, extensor plantar responses, dysarthria, and decreased vibratory sense and proprioception [1]. Most FA patients also present hypertrophic cardiomyopathy, cardiac dysfunction being the leading cause of death [2]. The disease is caused by mutations in a gene on chromosome 9, called *FXN* (for frataxin) or *X25* that results in decreased frataxin content or expression. In most patients, frataxin deficiency is caused by the presence of a GAA triplet expansion in the first intron of the *FXN* gene. This expansion is found in both alleles and compromises frataxin expression [3]. Around 4% of patients are compound heterozygotes for a GAA expansion and a *FXN* point mutation or deletion [4].

Decreased expression of frataxin is associated with mitochondrial dysfunction, iron and calcium imbalance, and increased oxidative stress. The function of frataxin and the mechanisms causing such cellular disturbances are not completely understood. Frataxin has been shown to localize to the mitochondria, where it regulates the activity of cysteine desulfurase, an enzyme required for the biosynthesis of iron–sulfur clusters [5]. Therefore, it is generally accepted that frataxin activates iron–sulfur biogenesis in eukaryotes. Nevertheless, iron–sulfur deficiency is not a universal consequence of frataxin deficiency, suggesting that the role of frataxin in iron–sulfur biogenesis is not essential [6]. Frataxin deficiency also causes mitochondrial iron overload, and different mechanisms have been proposed to explain this fact. First, compromised iron–sulfur biogenesis could interfere with iron sensing by Iron Regulatory Proteins, leading to constitutive activation of iron uptake [7–9]. Second, frataxin binds iron and presents ferroxidase activity, therefore, it could be involved in iron chelation and/or detoxification inside mitochondria [10]. Furthermore, large oligomers of frataxin are formed in vitro in the presence of iron, which could be involved in iron storage. Nevertheless, the in vivo relevance of such structures is controversial (reviewed in [11]). Third, an extramitochondrial form of frataxin has also been described that would bind and regulate Iron Regulatory Protein 1 [12].

Besides iron homeostasis and/or iron–sulfur biogenesis, other processes are altered in frataxin-deficient cells. Several observations suggest that oxidative stress could play a central role in FA. Hypersensitivity to oxidative damage has been observed in FA models and patients, and has been linked to deficient activation of the NRF2 pathway in response to oxidative insults. This pathway is required to induce the expression of antioxidant defenses [13], 14]. The central role of oxidative stress in FA is also highlighted by recent reports indicating that frataxin-deficient cells are hypersensitive to erastin, a drug that causes GSH depletion and is a well-known inducer of ferroptosis [15, 16]. Ferroptosis is a form of regulated cell death, which is triggered by the accumulation of lipid peroxidation products in membranes [17]. Frataxin deficiency has also been shown to impact different pathways related to mitochondrial function. These include the OXPHOS system [18], 19], mitochondrial calcium efflux [20], mitochondrial permeability pore opening [21], pyruvate dehydrogenase complex [22], and endoplasmic reticulum–mitochondria contacts [23], among others.

Several missense mutations in the frataxin coding region have been identified in FA patients, however, the in vivo consequences of these mutations have hardly been investigated. Galea and collaborators reviewed the consequences of the different frataxin mutations causing FA. Using structural modeling and a systematic literature review they predicted the consequences of these mutations on frataxin structure and function. They also compared clinical information from patients and concluded that there were no significant differences in mean age of onset between GAA-expansion homozygotes and compound heterozygotes carrying missense mutations [24]. This is consistent with observations indicating that the clinical phenotype of patients carrying the I154F mutation is similar to that of individuals homozygous for the GAA expansion [3]. The I154F mutation is one of the most studied frataxin missense mutations. Using cell-free systems and different cellular models, Li and collaborators analyzed the consequences of this mutation on the stability and catalytic activity of mature and intermediate frataxin proteoforms. They observed that I154F compromised the solubility of the intermediate frataxin form and resulted in low levels of mature frataxin. They also observed the presence of insoluble intermediate frataxin proteoforms in cells overexpressing this mutant version of frataxin [25]. From these observations it could be hypothesized that the presence of these insoluble frataxin proteoforms could contribute to the pathology. Besides, I154F mutation has also been reported to inhibit the interaction of frataxin with ISD11 [26] and also to decrease its ability to activate cysteine desulfurase [27].

In the present work we have analyzed the in vivo consequences of the I154F mutation by introducing an I151F substitution into mouse frataxin. This mutation is equivalent to the human I154F pathological mutation. We have observed that FXN^I151F^ homozygous mice present very low frataxin levels, neurological deficits and biochemical alterations which mimic those observed in patients with FA. Therefore, we can conclude that the pathological mechanisms of the I154F mutation are similar to those caused by the GAA triplet expansion and that the FXN^I151F^ mouse model is an excellent tool for analyzing the consequences of frataxin deficiency and for testing new therapies.

## Materials and methods

### *Generation of FXN*^*I151F*^* mice*

The investigation with experimental animals conforms to the National Guidelines for the regulation of the use of experimental laboratory animals from the Generalitat de Catalunya and the Government of Spain (article 33.a 214/1997) and was evaluated and approved by the Experimental Animal Ethical Committee of the University of Lleida (CEEA). FXN^I151F^ heterozygous mice (C57BL/6 J-*Fxn*^*em10(T146T,I151F)Lutzy*^/J) were obtained from the Jackson Laboratory, Bar Harbor, ME, USA (Stock Number 31922). To generate these HET mice, the CRISPR + guide targeted mutagenesis reagent was microinjected into C57BL/6 J zygotes. A founder female was crossed with C57BL/6 J to generate N1 HET mice which were also placed into mating to generate N2 HET mice. The incorporation of the I151F point mutation was verified by sequencing the *Fxn* gene. The mutant allele presented an ATC → TTC mutation in codon I151, which causes the expected I151F mutation. An additional silent mutation was present in codon T146 (ACC → ACT). The silent T146T mutation was co-introduced with the I151F mutation as a PAM blocker. Intercrosses of HET animals were performed to generate the WT, HET and FXN^I151F^ animals used in the present study. Genotyping of these mice was performed by sequencing a PCR product amplified from DNA extracted from tail biopsy specimens. The primers used were Fw: TTTCACACTTCCTGCCACCT; Rv: AGGCAGACAGCCGTAAAGTC. Animals were housed in standard ventilated cages at 12 h light/dark cycles and fed with a normal chow diet ad libitum. Animals were weighed weekly. For tissue isolation, animals were sacrificed by cervical dislocation at 21 or 39 weeks of age and dissected immediately. Isolated organs were snap‐frozen in liquid nitrogen and stored at -80 °C.

### Behavior analyses

The tests performed were Rotarod, Open Field, Hanging Wire, and Paw Print. All tests were conducted by the same investigator and under dark light conditions. The number of mice used in each analysis is indicated in Supplemental Table 1. Mice were subjected to Rotarod every two weeks from 15 to 39 weeks of age. Open Field, Hanging Wire, and Paw Print tests were performed at 21, 27, 33, and 39 weeks of age. The Rotarod test was performed in a LE 8200 Rotarod system (Panlab Harvard Apparatus). Animals were subjected to three trials in which, after 1 min at constant velocity (5 rpm), the rotarod was accelerated to 0.05 rpm/s. The latency to fall after acceleration was recorded and the average of the three trials was the value used. For the Open Field test, a mouse was placed in the center of the field and its movements were recorded during 5 min using Smart Video Tracking software (v2.5.21, Panlab Harvard Apparatus). The parameters measured were total distance travelled, entries in zones (crossings), and average speed. The Hanging Wire test was performed to assess forelimb grip strength and consisted in testing the ability of the mouse to hang on a horizontally positioned wire for 30 s. When the mouse fell from the wire, the latency to fall was recorded. Each mouse had three opportunities to achieve the 30 s objective. The best score of the three trials was the value used. Bedding material was placed underneath the wire to break the fall. For the Paw Print test, 55 cm long and 10 cm wide runway covered with laboratory paper was used. First, each mouse was placed on the runway during 30 s for habituation. Then, hind and fore paws were painted with pink and blue nontoxic paint, respectively, and the mouse was placed at the beginning of the runway and allowed to walk. The papers were scanned and the stride length of the animals was measured using ImageJ software.

**Table 1:** Proteins analyzed in the targeted proteomics analysis.

Protein Name	Abbrev	Uniprot	Comments
Citrate Synthase	CS	Q9CZU6	Component of the TCA cycle
Mitochondrial Aconitase	ACO2	Q99KI0	Component of the TCA cycle
Succinate Dehydrogenase flavoprotein subunit	SDHA	Q8K2B3	Component of OXPHOS complex II and the TCA cycle
Succinate Dehydrogenase iron–sulfur subunit	SDHB	Q9CQA3	Component of OXPHOS complex II and the TCA cycle
Cytochrome c	CYC	P62897	Electron carrier involved in OXPHOS
Cytochrome c1, heme protein	CY1	Q9D0M3	Component of OXPHOS complex III
Cytochrome b–c1 complex subunit 2	QCR2	Q9DB77	Component of OXPHOS complex III
Cytochrome c oxidase subunit 2	COX2	P00405	Component of OXPHOS complex IV
ATP synthase subunit alpha	ATPA	Q03265	Component of OXPHOS complex V
ATP synthase subunit beta	ATPB	P56480	Component of OXPHOS complex V
PDH E1 component subunit alpha	PDHA1	P35486	Component of the E1 PDH complex
Dihydrolipoyllysine–residue acetyltransferase	DLAT	Q8BMF4	E2 component of the PDH complex
Dihydrolipoyl dehydrogenase	DLDH	O08749	E3 component of the PDH complex
Stress-70 protein	GRP75	P38647	Mitochondrial Chaperone
60 kDa heat-shock protein	HSP60	P63038	Mitochondrial Chaperone
Superoxide dismutase [Cu–Zn]	SOD1	P08228	Cytosolic superoxide dismutase
Superoxide dismutase [Mn]	SOD2	P09671	Mitochondrial superoxide dismutase
Alpha-enolase	ENOA	P17182	Glycolytic enzyme
Beta-enolase	ENOB	P21550	Glycolytic enzyme
Gamma-enolase	ENOG	P17183	Glycolytic enzyme
Pyruvate kinase PKM	PKM	P52480	Glycolytic enzyme
Glyceraldehyde-3-phosphate dehydrogenase	GAPDH	P16858	Glycolytic enzyme

The abbreviations used throughout this manuscript and the Uniprot accession codes are indicated. Transitions used for detection of each protein are indicated in supplemental information. TCA: Tricarboxylic acid cycle

PDH: Pyruvate dehydrogenase

### Preparation of tissue homogenates

A tissue sample (20–100 mg) was cut into 2–3 mm^2^ pieces. All pieces were placed in 1.5 ml screw cap polypropylene tubes in the presence of lysis buffer consisting of 50 mM tris(hydroxymethyl)aminomethane (Tris) HCl pH 7.5 containing a protease inhibitor cocktail (Roche). For each 100 mg of tissue, 375 μl of lysis buffer were used. Glass beads (0.5–1.0 mm) were added to the mixture, which was then homogenized in a BioSpec Mini-Beadbeater. Following homogenization, SDS was added to a 4% final concentration. This homogenate was vortexed for 1 min, heated at 98ºC for 5 min, sonicated and subsequently centrifuged at 12,000 g for 10 min. Protein content in the supernatant was quantified using the BCA assay (Thermo Scientific). For fractionation of tissue homogenates, tissues were homogenized in the presence of a homogenization buffer consisting of 25 mM HEPES–KOH, pH 7.4, 100 mM NaCl, and protease inhibitor cocktail (Roche). These homogenates were subsequently centrifuged at 20,000 g for 20 min at 4 °C, yielding a soluble supernatant and an insoluble sediment. The supernatant was transferred to a new tube and SDS was added to reach a 4% final concentration. The sediment was washed three times with homogenization buffer and resuspended to the original volume using homogenization buffer containing 4% SDS. Both fractions were heated at 100 °C for 3 min and loaded on SDS-PAGE gels.

### Protein quantitation by targeted proteomics

Tissue homogenates (50 μg of protein) were precipitated with cold acetone (9 volumes) and resuspended in 1% sodium deoxycholate, 50 mM ammonium bicarbonate. Then, proteins were subjected to reduction by 12 mM DTT and alkylation by 40 mM IAM. Mass spectrometry grade trypsin (SOLu-Trypsin, Sigma) was added to a final enzyme:substrate ratio of 1:50. After overnight digestion at 37ºC, formic acid was added to precipitate sodium deoxycholate. The resulting peptide mix was purified and enriched using 100 μl Pierce C18 ZipTips. Eluted fraction from the C18 ZipTip was evaporated using a Concentrator Plus (Eppendorf) and peptides were resuspended in 3% acetonitrile plus 0,1% formic acid containing a heavy peptide standards mixture. Heavy peptides were obtained from JPT (SpikeTidesTM_L). All peptide samples were analyzed on a triple quadrupole spectrometer (Agilent 6420) equipped with an electrospray ion source. Chromatographic separations of peptides were performed on an Agilent 1200 LC system using a Supelco Bioshell A160 Peptide C18 column (1 mm × 15 cm). Peptides (up to 15 µg of protein digest) were separated with a linear gradient of acetonitrile/water, containing 0.1% formic acid, at a flow rate of 75 μl/min. A gradient from 3 to 60% acetonitrile in 45 min was used. The mass spectrometer was operated in multiple reaction monitoring mode. Transitions were obtained from peptide atlas, SRM atlas or Prosit [28] and imported into Skyline software [29], which was also used to analyze results. Once validated and optimized, the SRM assays were used to quantify all the peptides analyzed using scheduled SRM mode in a single run (retention time window, 120 s; cycle time, 1 s). Two injections (replicas) were performed per sample, and the light to heavy abundance ratio (raw L/H ratio) calculated for each peptide. A very high correlation in the raw L/H ratio between both replicas was observed (R = 0.99, Supplemental fig. 5). For calculating protein abundance, first the raw L/H ratio of each peptide in each replica was divided by the mean raw L/H ratio of all the peptides in the same replica. This was performed in order to correct for potential protein loss during sample preparation. Then, this value was divided by the average value of each peptide among all samples and replicas. This value was termed relative L/H. The final protein abundance value in a sample was obtained by calculating the average value among the relative L/H values from the different replicas and peptides corresponding to the same protein and sample. For most proteins analyzed, reproducibility between experimental replicas (different animals from the same experimental condition) was very high, as indicated by coefficients of variance below 10% (Supplemental Fig. 5B). Peptides and transitions analyzed are shown in. Supplemental Table 2.

### Western blot

After SDS–polyacrylamide gel electrophoresis, proteins were transferred to PVDF (Millipore, IPVH00010) or Nitrocellulose (Sigma_Aldrich, 10,600,093) membranes and blocked with I-block (ThermoFisher, T2015). The membranes were probed with the following primary antibodies: Frataxin (1:1000 Abcam, ab219414; 1:500 Abcam, ab175402; 1:500 Abcam, ab113691; 1:500 MyBioSource, mbs8245785), aconitase 2 (1:15,000 Sigma, HPA001097), lipoic acid (1:20,000 Calbiochem, 437,695) and OxPhos (1:20,000 Invitrogen, 458,099), α-Tubulin (1:50,000 Sigma, T5168). The detection was performed using peroxidase conjugated secondary antibodies. Image acquisition was performed in a ChemiDoc MP system from Bio-Rad. Membranes were stained with Coomassie brilliant blue or Ponceau for normalization, where each entire lane densitometry was used. When required, data was analyzed by ImageLab software (Bio-Rad). Apparent MW was calculated using Precision Plus Protein Standards (Bio-Rad) and ImageLab software using the point-to-point semi-log interpolation method.

### Enzyme activities

A tissue sample was cut into 2–3 mm^2^ pieces. All pieces were placed into tubes containing nondenaturing lysis buffer consisting of Tris–HCl 50 mM pH 7.4, protease inhibitor cocktail (Roche), and sodium citrate 2.5 mM. Glass beads (0.5–1.0 mm) were added and tissues were homogenized using a BioSpec Mini-Beadbeater. Then, Triton X-100 was added to a final concentration of 0.5%. Homogenized tissues were centrifuged at 13,000 rpm at 4ºC during 5 min and the supernatants were placed into new tubes. Aconitase activity was measured in Tris–HCl 50 mM pH 7.4, containing 1 mM sodium citrate, 0.2 mM NADP, 0.6 mM manganese chloride, and 0.25 units of isocitrate dehydrogenase (Sigma-Aldrich, I2002). NADPH formation was measured at 340 nm during 120 s. Citrate synthase activity was measured with a coupled assay to reduce 5,5′-dithiobis-(2-nitrobenzoic acid) (DTNB). Briefly, tissue extracts were added to Tris–HCl 100 mM pH 8.1 with 0.4 mg/ml DTNB and 10 mg/ml Acetyl-CoA. Absorbance was measured at 412 nm during 120 s. Then, 8.5 mg/ml of oxaloacetate were added to the cuvette and the absorbance was measured again at 412 nm during 120 s for the detection of reduced DTNB. Values are presented as a ratio of aconitase activity *versus* citrate synthase activity.

### Quantitative real-time PCR

For qRT-PCR analysis, 50–100 µg of tissue sample was homogenized with 1 mL of TRIzol™ Reagent (Invitrogen, 15,596,018) using an IKA® T10 basic homogenizer. RNA was extracted following manufacturer’s instructions. For each sample, 1 µg of total RNA was converted into cDNA with the iScript cDNA Synthesis Kit (Bio-Rad, 1,708,891) and 50 ng of cDNA were used in each reaction. The assays were performed in a CFX96 Real-Time System (Bio-Rad) using TaqMan® 2X Universal PCR Master Mix (Applied Biosystems, 4,304,437). Real-Time PCR was performed using TaqMan probes: Sdha (Mm01352366_m1), Sdhb (Mm00458272_m1), Ndufb8 (Mm00482663_m1), Aco2 (Mm00475673) and Gapdh (Mm99999915_g1). Gapdh was used as an internal control. Quantification was completed using Bio-Rad CFX Manager real-time detection system software (version 3.1, Bio-Rad). Relative expression ratios were calculated on the basis of ΔCp values with efficiency correction based on multiple samples.

### Frataxin overexpression

HEK 293 T cells were plated in 35 mm collagen coated plates at a density of 3·10^5^ cells/plate. After 20 h, cells were washed once with optiMEM™ medium (Gibco, 31,985–047) and transfected with a pCMV6-Entry vector containing untagged mouse Fxn ORF (OriGene, NM_008044) using 10 µM of Polyethylenimine in optiMEM™ medium. Cells were incubated for 1 h at 37ºC and then the plasmid was removed and cells were cultured with DMEM (Gibco, 41,966–029) supplemented with 10% Fetal Bovine Serum. After 24 h, cells were washed once with cold PBS and lysed in 200 µl lysis buffer consisting of 125 mM TRIS–HCl pH 7.5, 2% SDS and protease inhibitor cocktail (Roche). Lysates were heated at 100 °C for 3 min and loaded on SDS-PAGE gels.

### Statistical analysis

Statistical analyses were performed using GraphPad Prism (version 8). The two-tailed Student’s t test was used to assess the significance of the differences between protein and mRNA content data. In mouse analyses, differences between groups were assessed by two-way ANOVA with Benjamini, Krieger and Yekutieli post hoc test. The p values lower than 0.05(*), 0.01(**) or 0.001(** *) were considered significant.

## Results

### Generation of frataxin I151F knock-in mice

**Fig. 1:**
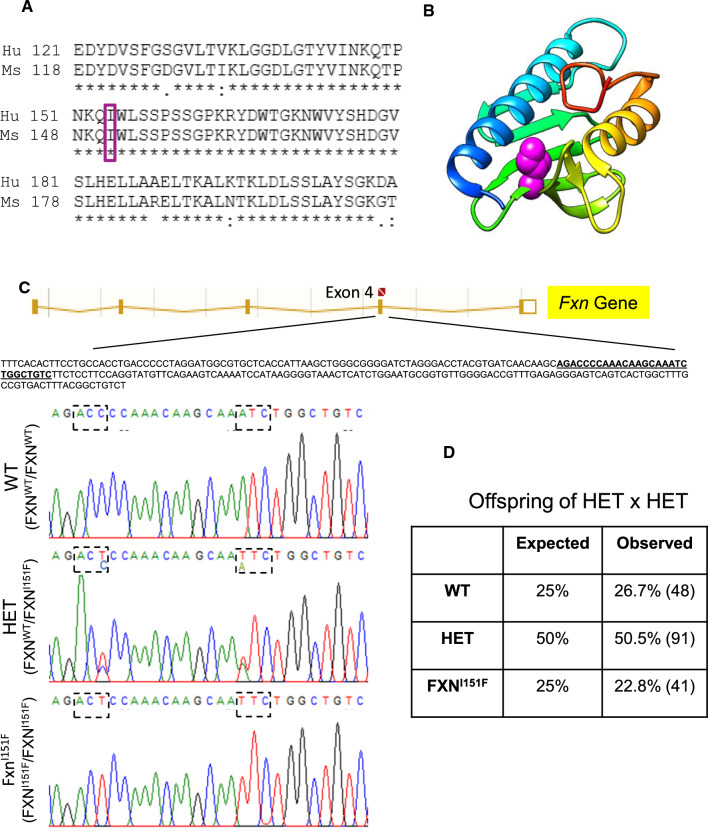
Generation of the FXN^I151F^ model. **A** Alignment of the C-terminal region from human (Hu, Uniprot Q16595) and mouse (Ms, Uniprot O35943) frataxin protein sequences. Amino acid I151 in mice corresponds to I154 in humans (boxed). **B** ribbon representation of human frataxin (pdb code 3s4m) showing the position of I154 (spacefill, in violet). Ribbons are colored according to sequence, from dark blue (N-terminal) to red (C-terminal). Molecular graphics were performed with the UCSF Chimera package. **C** DNA sequence analyses of WT, HET, and FXN^I151F^ mice. The top panel shows a schematic diagram of the *Fxn* Gene. The amplified sequence used in genotyping assays includes exon 4 and is indicated. The region containing the mutations is highlighted and shown below. Dashed boxes indicate the ACC → ACT (silent) and ATC → TTC (I151F) mutations introduced by gene editing into the frataxin gene. **D** Genotyping of litters from HET intercrosses

FXN^I151F/wt^ heterozygous mice (C57BL/6J-Fxn^em10(T146T,I151F)Lutzy/J^) were obtained from the Jackson Laboratory (Stock Number 31922). To generate these mice, CRISPR/Cas9 was used to introduce the ATC→TTC mutation into codon I151 of murine *Fxn. *The resulting I151F substitution is equivalent to the human pathogenic I154F missense mutation (Figure 1A). An additional silent mutation is present in codon T146 (ACC→ACT). This change was deliberately co-introduced with the desired mutation in order to destroy the guide dependent protospacer adjacent motif (PAM) recognition site. Inter-crossing of these mice resulted in progeny of all possible genotypes. Of 180 mice born, 48 (26,7%) were WT mice (FXN^wt/wt^ homozygous for wild type FXN), 91 (50,5%) were HET mice (FXN^I151F/wt^, heterozygous for wild type FXN and FXNI151F), and 41 (22,8%) were FXN^I151F^ mice (FXN^I151F/I151F^ , homozygous for the FXNI151F allele) (Figure 1D). Therefore, progeny was within the expected mendelian ratios indicating that FXN^I151F^ mice were viable.

### Frataxin protein levels are markedly reduced in FXN^I151F^ mice

**Fig. 2:**
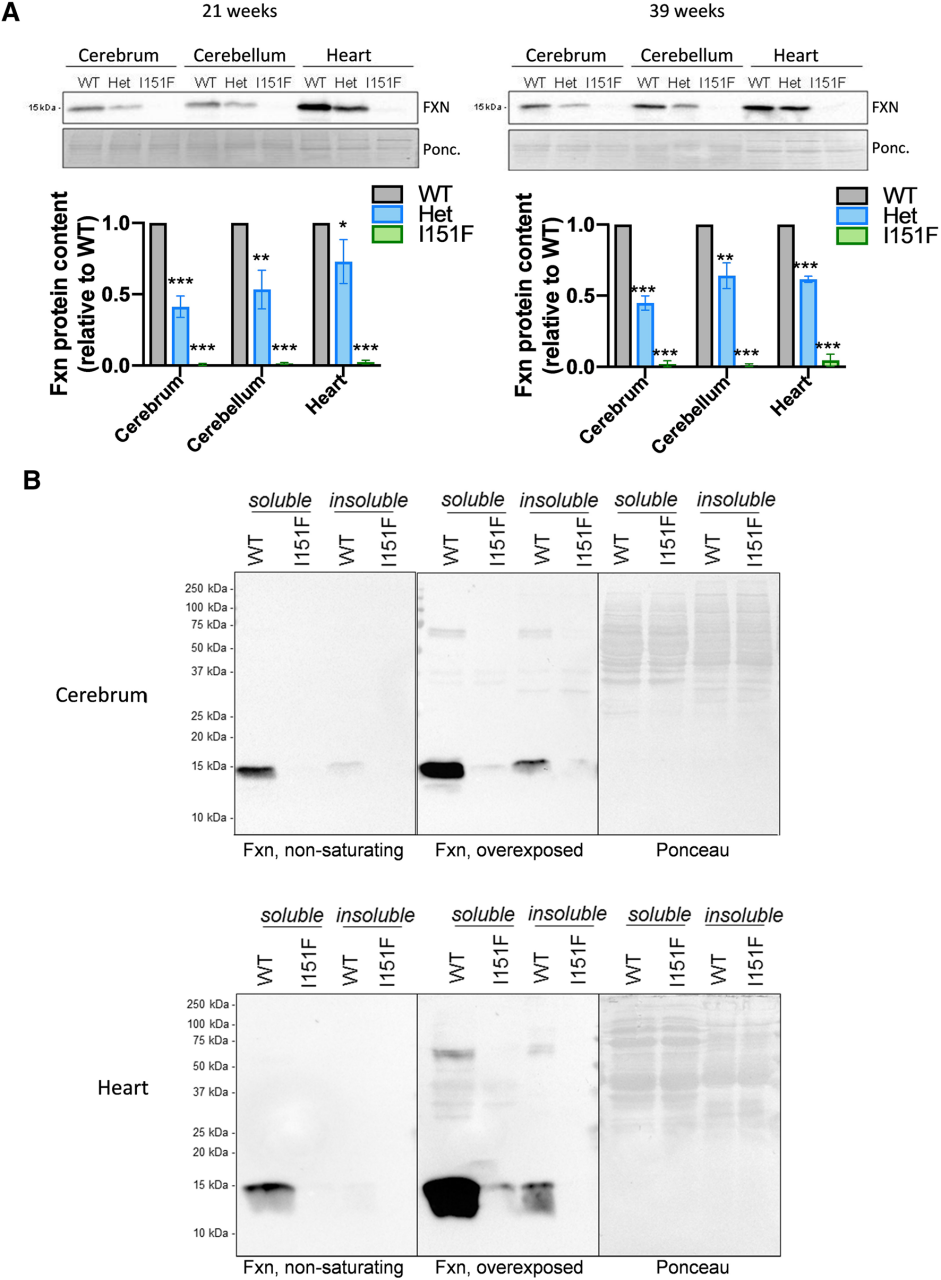
FXN^I151F^ mice present decreased frataxin content. **A** Relative mature frataxin (15 kDa) content analyzed by western blot in cerebrum, cerebellum, and heart homogenates from WT, HET, and FXN^I151F^ mice. **B** Cerebrum and heart homogenates from 21-week-old WT and FXN^I151F^ mice were prepared under native conditions and the soluble and insoluble protein fractions separated by centrifugation. Representative images from frataxin western blot membranes under nonsaturating or overexposed conditions are shown. Whole membrane is shown to indicate the absence of insoluble full-length or intermediate frataxin proteoforms in FXN^I151F^ samples

To determine the effect of the I151F mutation on frataxin content, we measured the levels of this protein in cerebrum, cerebellum and heart from 21 and 39-week-old WT, HET and FXN^I151F^ mice. By western blot (using monoclonal antibody Ab219414), we observed that the content of mature frataxin in HET mice was approximately 50% of that observed in WT mice, while less than 6% of mature frataxin content was observed in FXN^I151F^ mice (Fig. 2A and supplemental Figure 1B). We also analyzed the frataxin content in homogenates from spinal cord, dorsal root ganglia (DRG), liver, pancreas and skeletal muscle. These analyses confirmed that the I151F mutation causes a marked loss of frataxin content in all mouse tissues (Supplemental Figure 1). In order to exclude the possibility that the decreased frataxin signal in the western blot was caused by a loss of epitope recognition by the monoclonal antibody employed, three additional antibodies were used to detect frataxin in cerebrum homogenates. These were two polyclonal antibodies raised against full-length frataxin (Mbs8245785 and Ab175402), and a monoclonal antibody (Ab113691) raised against a peptide within aa 1-120 from human frataxin (thus not including I154). Also, we overexpressed murine frataxin in HEK293 cells in order to identify the migration pattern of mature, intermediate and frataxin proteoforms. These results are shown in supplemental figure 2A. Three major bands can be overserved in overexpressing cultures frataxin: the slower migrating band has an apparent molecular weight of 26.6 kDa, and corresponds to the precursor form (theoretical MW 22.9 kDa); the intermediate form has an apparent MW of 18.1 kDa (theoretical MW 18.6 kDa); the mature form has an apparent MW of 15.3 kDa (theoretical MW 14.4 kDa). Regarding frataxin detection in cerebrum homogenates with the different antibodies used, all of them were able to detect a band migrating at 15 kDa in WT samples. This band disappeared in I151F samples, confirming that the mature frataxin content in FXN^I151F^ mice is very low. The Mbs8245785 and Ab113691 antibodies recognized bands in the 20-26 kDa range, but these bands may be caused by cross-reaction of the antibody with unknown proteins, as they were not detected by antibodies Ab219414 and Ab175402. To further confirm the frataxin decrease in FXN^I151F^ mice, we used a mass spectrometry-based targeted proteomics SRM approach to detect frataxin in tissues collected from WT and FXN^I151F^ mice. This approach was based on the detection of proteotypic peptides from frataxin in trypsin-digested tissue homogenates, which were analyzed in a liquid chromatograph coupled to a triple quadrupole mass spectrometer (proteotypic peptides are those peptides in a protein that are most likely to be observed by mass spectrometry and which are unique to that protein). We selected the four peptides with higher ESS score in peptideatlas server (ESS provides an estimation of the proteotypicity of each peptide) and for each one of these we selected the three most frequently observed transitions. These transitions are indicated in Supplemental Figure 2B. All these peptides are present in both mature, precursor and intermediate forms of frataxin. Using this approach, we were able to detect a signal in heart homogenates eluting at minute 22.7, corresponding to the 2+ to y7+ transition of the LGGDLGTYVINK peptide. We could not detect this signal in cerebrum or cerebellum homogenates, probably because frataxin is less abundant in the nervous system than in heart (as observed in figure 2A). None of the other peptides analyzed were detected. It is worth mentioning that frataxin is not easily detected by mass spectrometry as according to peptide atlas frataxin has been observed 386 times, while mitochondrial aconitase has been observed 26660 times. In order to confirm that the signal observed in heart homogenates corresponded to the LGGDLGTYVINK peptide, an isotopically labeled heavy version of the peptide was purchased and included in the analysis as an internal standard. This heavy form presented the same retention time as the light form. Moreover. the light form was not detected in heart from FXN^I151F^ mice (Supplemental figure 2C), further confirming that frataxin was markedly decreased in these animals. Due to its low intensity, we were not able to accurately quantify the residual frataxin present in mutant mice, but we could estimate that it was below 5% of WT values. This value is consistent with the western blot data and confirms that frataxin is markedly decreased in FXN^I151F^ mice. These results also indicate that intermediate or precursor proteoforms are not detected in neither WT or FXN^I151F^ mice.

### Insoluble intermediate frataxin proteoforms are not detected in FXN^I151F^ mice

The presence of an insoluble intermediate frataxin proteoform has been reported in cells overexpressing human I154F frataxin [25]. Results presented in the previous section suggest that this form may not be present in FXN^I151F^ mice, as the denaturing conditions used for protein extraction in these assays should solubilize such intermediate insoluble forms. Nevertheless, in order to confirm this point, we analyzed the potential presence of this insoluble form under the same conditions used to detect human insoluble FXNI154F. To this purpose, we prepared tissue homogenates under native conditions, and we separated the soluble protein fraction from the insoluble protein fraction by centrifugation. After solubilizing the insoluble fraction in a buffer containing 4% SDS (a concentration high enough to solubilize the previously reported I154F insoluble intermediate frataxin form) both fractions were loaded on SDS-PAGE gels and frataxin proteoforms detected by western blot (antibody Ab219414). Membranes were overexposed in order to detect any potential intermediate or high molecular weight forms of frataxin. In these membranes, we could detect the presence of some high molecular weight bands in WT homogenates (Figure 2B). Nevertheless, these high molecular weight forms represented less than 1% of the chemiluminescent signal from the mature form, and were also decreased in homogenates from FXN^I151F^ mice. Therefore, we can exclude the presence of an insoluble intermediate frataxin proteoform in mice harboring the I151F mutation.

### FXN^I151F^ mice present decreased weight gain

**Fig. 3:**
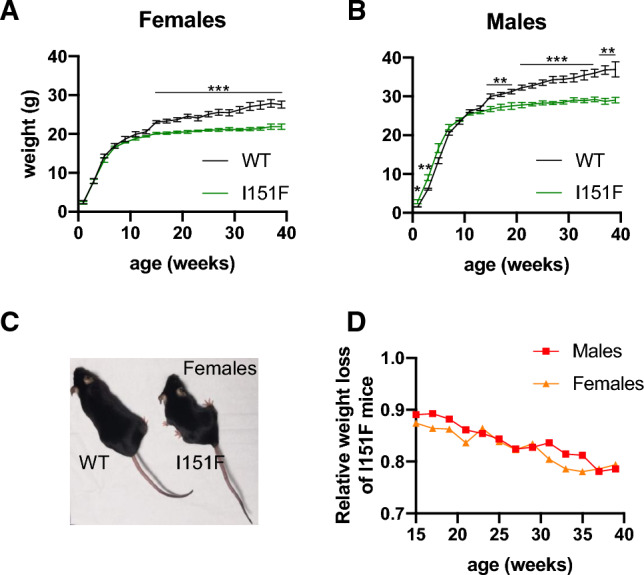
FXN^I151F^ mice present decreased weight gain. **A** and **B** Body weight of WT and FXN^I151F^ mice up to 39 weeks of age. **C** Representative image from WT and FXN^I151F^ female mice. Values are shown as mean ± SEM. **D** Average weight loss of FXN^I151F^ mice relative to WT mice of the same age. Number of animals used in each analysis is indicated in supplemental table 1

Body weight of WT and FXN^I151F^ mice was measured every two weeks from birth. Weight gain was similar in WT and FXN^I151F^ mice until 10 weeks of age. From that age on, weight gain was lower in FXN^I151F^ mice than in WT mice. Therefore, significant weight differences were observed between WT and FXN^I151F^ mice from 15 weeks of age onward (Figure 3A and B). The relative weight difference between WT and FXN^I151F^ mice was progressive and was similar in both females and males. At 39 weeks of age, FXN^I151F^ mice presented on average a 23% decrease in weight when compared with WT mice (Figure 3C and D). HET mice did not present any weight loss (Supplemental Figure 3A).

### FXN^I151F^ mice exhibit neurological deficits

**Fig. 4:**
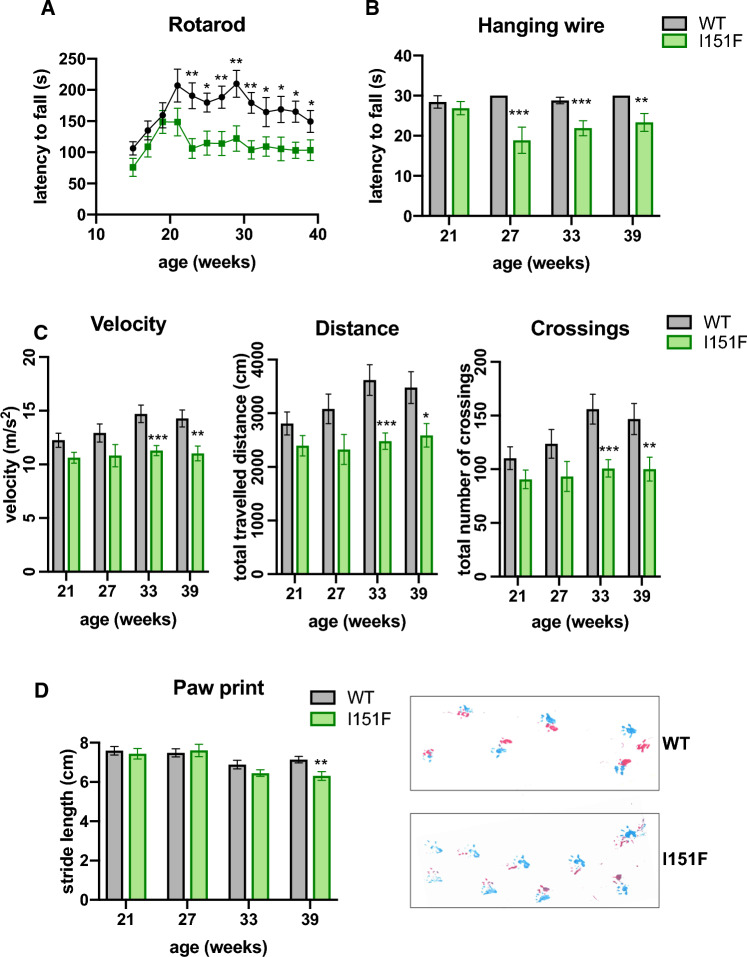
Neurological deficits in FXN^I151F^ mice. Rotarod, Open field, hanging wire, and Paw print analysis in WT and FXN^I151F^ animals; **A** Rotarod test in mice up to 39 weeks of age. FXN^I151F^ animals presented a decreased latency to fall from 21 weeks onward. **B** Hanging wire test in mice up to 39 weeks of age. FXN^I151F^ animals presented a decreased latency to fall from 27 weeks onward. **C** Open field test. FXN^I151F^ mice showed significant decline in velocity, total distance traveled, and number of crossings from 33 weeks onward. **D** Gait footprint analysis revealed abnormalities in walking patterns in FXN^I151F^ mice, which displayed significantly reduced stride length at 39 weeks of age. Representative walking footprint patterns are shown, in which pink and blue marks represent hind and fore paws respectively. Values are shown as mean ± SEM. Number of animals used in each analysis is indicated in Supplemental Table 1

To determine whether frataxin deficiency caused by the I151F mutation impacted the behavior of FXN^I151F^ mice, we subjected these mice to several motor behavioral tasks. WT mice were also analyzed as a control. The number of mice used for each analysis is summarized in Supplemental Table 1. The motor coordination ability was assessed on a rotarod treadmill. This analysis was performed every two weeks, from 15 to 39 weeks of age. As shown in figure 4A, FXN^I151F^ mice showed decreased coordination ability compared with WT. Differences were statistically significant from week 23 onward. Forelimb strength was tested using a hanging-wire test, where mice were assessed for their ability to hang on a horizontal wire by their forepaws. Analyses were performed every six weeks (starting at week 21). FXN^I151F^ mice fell off the wire quicker than WT mice. Statistically significant differences were obtained for 27-, 33-, and 39-week-old mice (Figure 4B). Locomotor activity tests were performed using an open-field beam-breaker activity monitor. These analyses were performed every six weeks (starting at week 21). FXN^I151F^ mice exhibited significantly reduced average velocity, ambulatory distance (total distance covered by the mice within a specific time), and number of crossings than WT mice (Figure 4C). Finally, we assessed gait ataxia using paw print analysis every six weeks (starting week 21). Significant differences between FXN^I151F^ and WT mice were detected in 39-week-old mice, but not before. At this age, FXN^I151F^ mice displayed reduced hind and front limb stride length compared with WT. This suggests that FXN^I151F^ mice present a progressive ataxic gait (Figure 4D). Overall, the results presented in this section indicate that FXN^I151F^ mice present progressive neurologic defects that are not observed before 23 weeks of age. Finally, in order to explore the effect of the I151F mutation in heterozygosis, we analyzed the performance of HET mice in an open field test. No significant differences were observed in the open field test between WT and HET mice, neither in velocity, distance travelled, nor number of crossings (Supplemental Figure 3B).

### Analysis of frataxin-related proteins by targeted proteomics

**Fig. 5:**
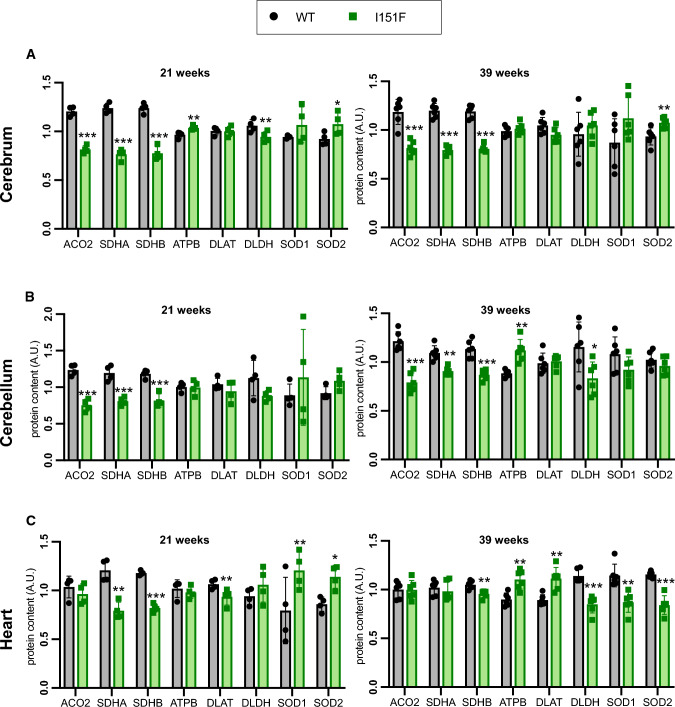
Analysis of the content of frataxin-related proteins by targeted proteomics. Histograms A to C show the relative content (in arbitrary units) of the proteins that present the most marked changes by targeted proteomics in either cerebrum, cerebellum, or heart from 21- and 39-week-old WT and FXN^I151F^ mice. Data are represented as mean ± SD from four (21-week) or six (39-week) mice. Significant differences p values < 0.05(*), 0.01(**), or 0.001(** *) between WT and FXN^I151F^ mice are indicated

We were interested in analyzing the biochemical consequences of frataxin deficiency in nervous and cardiac tissues before (21-weeks) and after the onset of neurological defects (39-weeks). With this purpose, we decided to focus on several proteins or pathways that have been related to frataxin. In this regard, frataxin deficiency has been described to cause loss of iron-sulfur containing proteins [30], decreased function of the OXPHOS system [19], changes in the content of superoxide dismutases [31], and decreased content of the pyruvate dehydrogenase component PDH A1 [22]. Also, frataxin has been reported to interact with components of the iron-sulfur biosynthesis machinery [32], the OXPHOS system [33], and with the mitochondrial chaperone GRP75 [26, 34]. Therefore, in order to explore these pathways in FXN^I151F^ mice, we set up a targeted proteomics method to analyze the content of proteins related to these pathways in tissues collected from WT and FXN^I151F^ mice. We used an SRM-based targeted proteomics approach in which proteotypic peptides from each protein were detected in a LC-triple quadrupole mass spectrometer. This approach allows the quantitation of several proteins in multiple samples with high reproducibility. The proteins analyzed are listed in Table 1. They consisted of two components of the tricarboxylic acid cycle (CS and ACO2), several components of the OXPHOS system (SDHA, SDHB, CY1, QCR2, COX2, ATPA, and ATPB), two mitochondrial chaperones (HSP60 and GRP75), three components of the Pyruvate dehydrogenase complex (PDHA1, DLAT and DLDH), and two superoxide dismutases (SOD1 and SOD2). Three glycolytic enzymes were also included in the analysis (GAPDH, PKM, and Enolases A, B, G) in order to analyze a potential imbalance between respiratory and glycolytic pathways and/or between mitochondrial and cytosolic pathways. Two of the proteins analyzed contain iron-sulfur clusters (ACO2 and SDHB), while two of them (CYC and CY1) contain heme groups. The relative content of these 21 proteins was analyzed in cerebrum, cerebellum, and heart from WT and FXN^I151F^ mice, sacrificed at 21 or 39 weeks of age (in the case of the enolases, ENOG only in nervous systems and ENOB only in heart). Figure 5A-C summarizes the most relevant findings while Supplemental Figure 4 contains the results from all the proteins analyzed. It can be appreciated that the most affected proteins were ACO2 and the two components of the OXPHOS complex II (SDHA and SDHB). These proteins showed a marked decrease in cerebellum and cerebrum from FXN^I151F^ mice, both at 21 and 39 weeks. In heart, we could only observe loss of complex II in 21-week-old mice, but not in 39-week-old mice, while ACO2 content was not altered in heart at any age. Some other components of the OXPHOS system presented minor changes: the complex III components QCR2 and CY1 were decreased in cerebrum (21 weeks), cerebellum (21 and 39 weeks), and heart (21 weeks); ATPA and ATB were increased in 39-week-old cerebellum and heart. A second group of proteins showing changes in their content were the antioxidant enzymes SOD1 and SOD2. In heart, both enzymes were induced in 21-week-old mice, while their levels decreased in 39-week-old mice. Some induction of SOD2 was also observed in cerebrum at 21 and 39 weeks. Regarding PDH components, induction of both PDHA1 and DLAT was found in 39-week-old heart. In contrast, DLDH (the E3-component of the PDH complex) was decreased in 39-week-old heart and cerebellum. No major changes were observed in the content of the mitochondrial chaperones analyzed, nor of the glycolytic enzymes. The net representation shown in Supplemental Figure 4 allows a comparison of the relative content of the proteins (ratio FXN^I151F^ /WT) at 21 and 39 weeks of age. Overall, these results indicate that frataxin deficiency does not cause a general loss of mitochondrial proteins. Instead, it causes specific changes in the content of certain proteins. Also, the net representation indicates how these changes evolve as mice age. In this regard, cerebrum and cerebellum do not experience major differences in the FXN^I151F^ /WT protein ratio between 21-week-old and 39-week-old mice. Therefore, biochemical alterations precede the neurological defects described in the previous section. In contrast, the heart shows differences in the FXN^I151F^ /WT protein ratio between 21- and 39-week-old mice, suggesting that the consequences of frataxin loss in this tissue are progressive. Finally, we analyzed the content of some representative proteins in cerebrum, cerebellum, and heart from HET mice. The proteins analyzed were CS, ACO2, SDHB, ATPB, SOD2, DLAT, GRP75, and PKM. This group includes representative proteins that experienced changes in the mutant mice (ACO2, SDHB, ATPB and SOD2), and also proteins that did not experience any changes, but are representative of different pathways/functions. These proteins were analyzed in cerebrum, cerebellum, and heart from 39-week-old mice by targeted proteomics. None of these proteins presented relevant changes in their content comparing HET and WT animals (results shown in Supplemental Figure 3). A slight decrease in the amounts of CS and SDHB was observed in the cerebellum of HET mice, but these changes were lower than those observed in FXN^I151F^ mice and/or were not observed in cerebrum and heart.

### FXN^I151F^ mice present lower aconitase activity

**Fig. 6:**
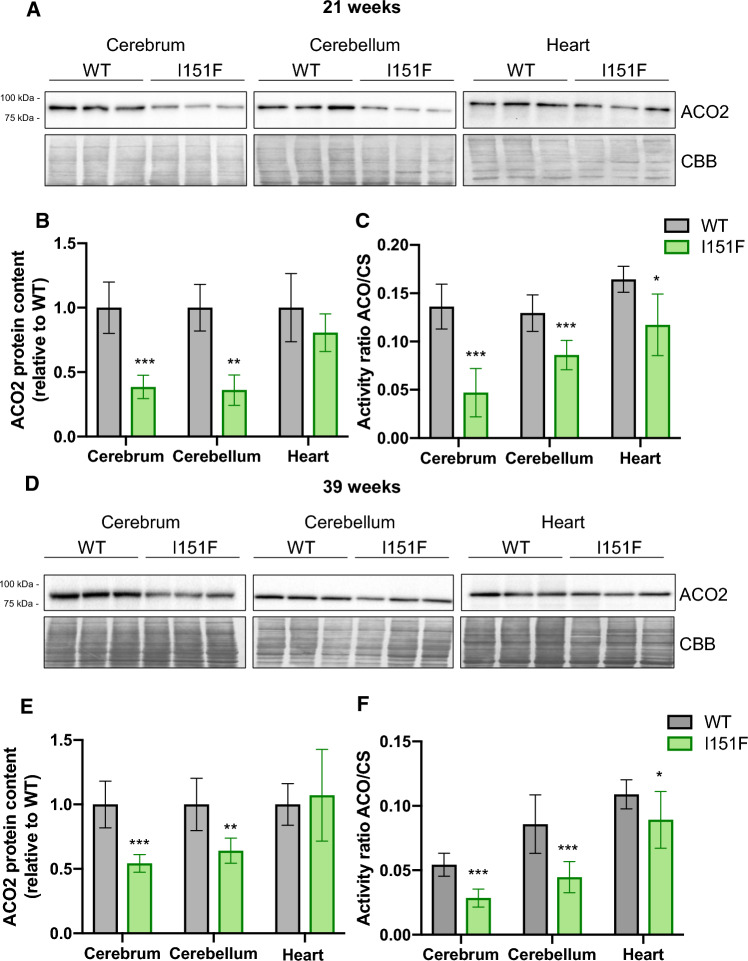
Aconitase content and activity in WT and FXN^I151F^ mice. ACO2 content was analyzed by western blot in cerebrum, cerebellum, and heart homogenates from 21-week (**A**) and 39-week (**D**) WT and FXN^I151F^ mice. Representative western blot images are shown in **A** and **D**. Samples from three different mice were loaded on gels. Histograms **B** and **E** represent the mean ± SD from at least four independent measurements; **C** and **F** Aconitase to Citrate synthase activity ratio (ACO/CS) was measured in cerebrum, cerebellum, and heart homogenates from 21-week **C** and 39-week **F** WT and FXN^I151F^ mice. Data represent the mean ± SD from at least five different mice. Significant differences between WT and FXN^I151F^ mice are indicated (p values < 0.05(*), 0.01(**) or 0.001(** *)

In order to validate the results of the targeted proteomics analysis, we decided to measure the content, activity, and/or expression levels of those proteins most markedly altered in the proteomic analysis. We first focused on ACO2, a mitochondrial iron-sulfur enzyme, which converts citrate to isocitrate in the tricarboxylic acid cycle. As this enzyme is one of the most abundant iron-sulfur containing proteins, it is commonly used as an indicator of the status of iron-sulfur centers in the cell. Therefore, we analyzed its content by western blot and its activity by spectrophotometry. These analyses were performed in cerebrum, cerebellum, and heart homogenates from 21-week and 39-week-old mice. ACO2 is expected to account for most of the aconitase activity in these tissues as it represents between 80 and 90 % of all aconitase content according to the PaxDb database. Western blot analysis confirmed the results observed in the targeted proteomic analysis, as the results obtained were similar to those obtained in the previous section: a marked decrease was observed in the nervous system, while in heart tissue, no significant differences were observed between WT and FXN^I151F^ mice (Figure 6). Regarding activity, we measured the ratio between aconitase and citrate synthase activities (ACO/CS ratio), which was used as a control for non-iron-sulfur mitochondrial enzyme. We observed a significantly decreased ACO/CS activity ratio in the three tissues tested, with more marked decreases in cerebrum and cerebellum than in heart (Figure 6). Overall, the results obtained confirm that aconitase content and activity is more affected in the nervous system than in heart. They also suggest that decreased aconitase activity is mostly due to decreased ACO2 protein content, although the presence of some inactivated protein cannot be excluded (as in some of the conditions analyzed, loss of activity is slightly higher than loss of protein content).

### FXN^I151F^ mice present decreased content of OXPHOS complexes I and II

**Fig. 7:**
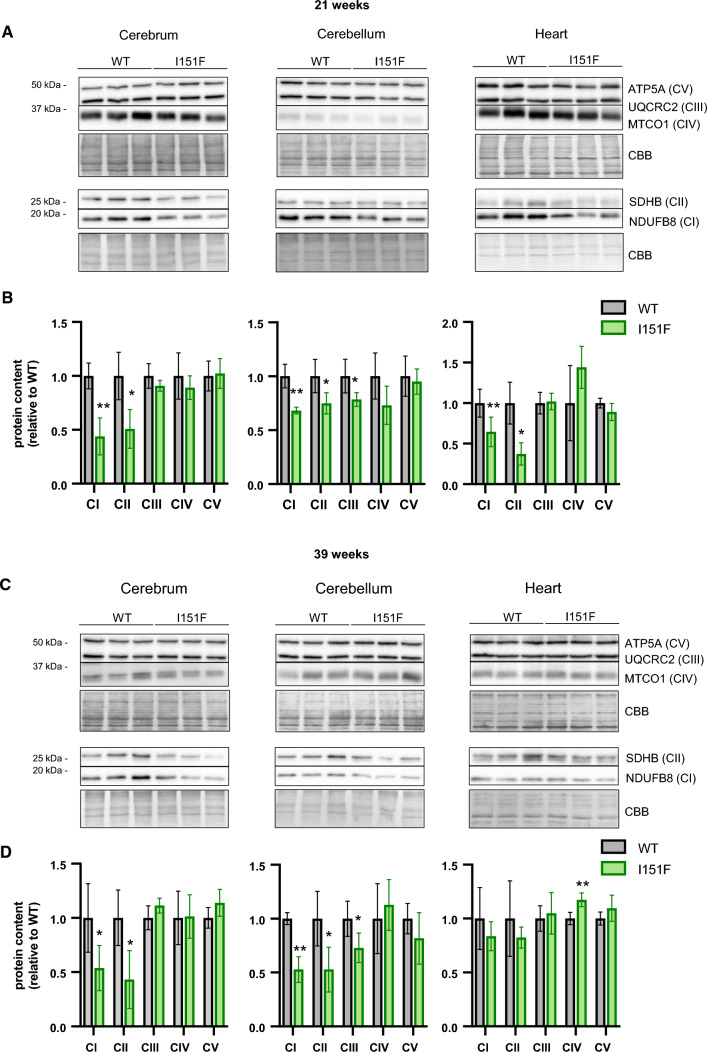
Western blot analysis of components of the OXPHOS system. The components indicated of the mitochondrial OXPHOS system were analyzed by western blot in cerebrum, cerebellum, and heart homogenates from 21-week (**A**) and 39-week (**C**) WT and FXN^I151F^ mice. Representative western blot images are shown in **A** and **C**. Samples from three different mice were loaded on gels. Histograms **B** and **D** represent the mean ± SD from at least four independent measurements. Significant differences between WT and FXN^I151F^ mice are indicated (p values < 0.05(*), 0.01(**), or 0.001(** *)

The targeted proteomics analysis also revealed changes in the content of several components of the OXPHOS system, notably complex II. In order to confirm these results with an alternative approach, we used western blot to analyze the content of five components of the complexes I to V (one protein per complex) in cerebrum, cerebellum, and heart from 21- and 39-week-old mice. These proteins were NDUB8 from complex I, SDHB (complex II), QCR2 (complex III), COX1 (complex IV), and ATPA (complex V). None of the complex I components had been included in the targeted proteomics analysis as they could not be properly quantified. The results obtained are shown in Figure 7. It can be observed that FXN^I151F^ mice present a marked loss of NDUB8 from complex I and SDHB from complex II in all tissues analyzed (except heart at 39 weeks), and that this decreased content was already observed at 21 weeks of age. The results from SDHB are similar to those found in the targeted proteomics analysis, confirming the validity of the data obtained. Regarding complexes III to V, a small decrease in QCR2 content (from complex III) could be appreciated in cerebellum, as previously observed in the targeted proteomics approach. No changes in ATPA content were observed by western blot analysis. Overall, the results obtained by the targeted proteomics approach and the western blot analysis indicate that FXN^I151F^ mice present a deficiency in complex I and II components of the OXPHOS system, especially in the nervous system, while the components of the other complexes do not present major differences.

### mRNA levels from NDUFB8, SDHA, SDHB, and ACO2

**Fig. 8:**
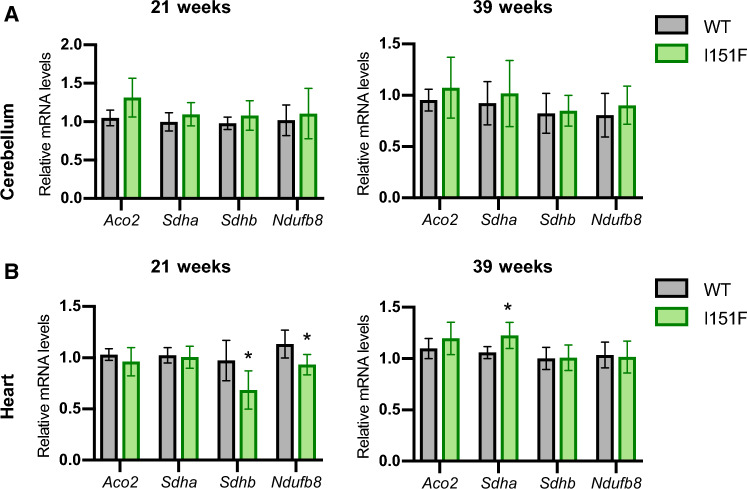
Analysis of mRNA expression by qPCR. Histograms show the relative mRNA content of the genes indicated in cerebellum (**A**) or heart (**B**) from 21- and 39-week-old WT and FXN^I151F^ mice. Data are represented as mean ± SD from six mice. Significant differences p values < 0.05(*), 0.01(**), or 0.001(***) between WT and FXN^I151F^ mice are indicated

In order to discern whether the decreased content of NDUFB8, SDHA, SDHB, and ACO2 proteins was caused by lowered gene expression or by other mechanisms, the mRNA levels of these genes were analyzed by qPCR (Figure 8). We performed this analysis in cerebellum and heart tissues from 21- and 39-week-old mice. In cerebellum, no changes were found in the mRNA levels of these genes when comparing WT and FXN^I151F^ mice. This result indicates that changes in protein content in this tissue are caused by posttranscriptional mechanisms. Regarding heart, a decrease in SDHB and NDUFB8 mRNA levels was observed in 21-week-old animals. Therefore, the decreased content of these proteins in heart can be attributed to a lowered gene expression. Indeed, in 39-week-old mice, the mRNA levels of both proteins are restored, as observed from protein content. In contrast, SDHA expression is not altered in 21-week-old mouse heart (despite lower protein content). Therefore, loss of SDHA in heart is also caused by posttranscriptional mechanisms. Overall, these results suggest that the mechanisms causing alterations in protein content may be tissue specific, and include transcriptional and posttranscriptional mechanisms.

### Biosynthesis of lipoic acid is not compromised in FXN^I151F^ mice

**Fig. 9:**
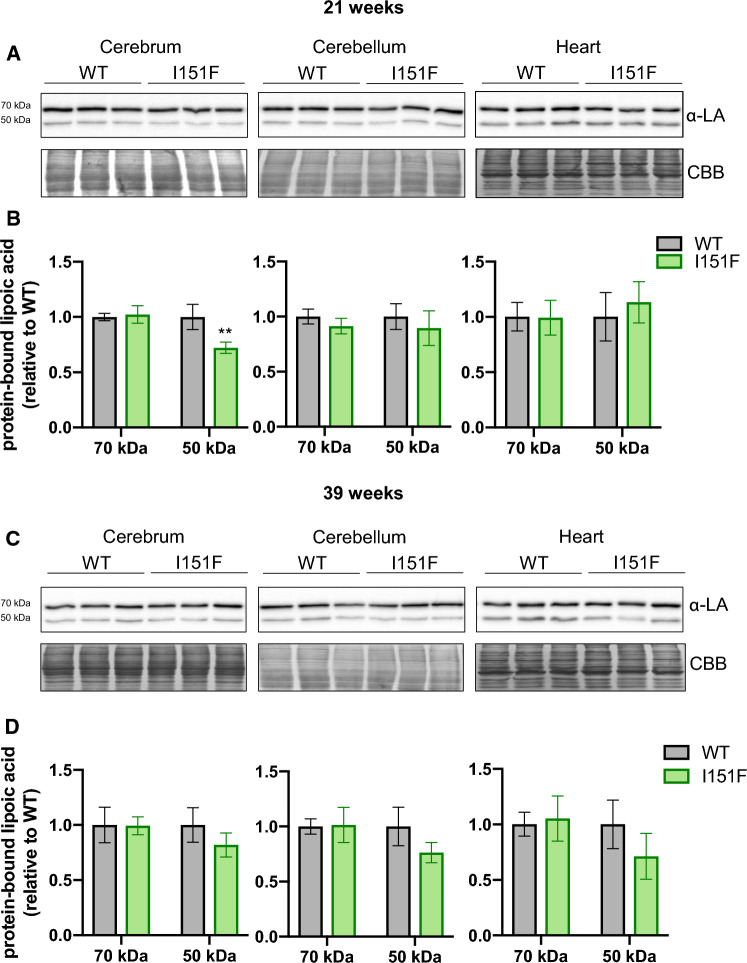
Western blot analysis of protein-bound lipoic acid. Protein bound lipoic acid was analyzed using lipoic acid (LA) antibodies in cerebrum, cerebellum and heart homogenates from 21-week (**A**) and 39-week (**C**) WT and FXN^I151F^ mice. Representative western blot images are shown in **A** and **C**. Samples from three different mice were loaded on gels. The 70 kDa band corresponds to lipoic acid bound to DLAT, while three lipoic acid containing proteins migrate at 50 kDa (DLST, E2 from the branched chain alpha-ketodehydrogenase complex, and PDH-X). Histograms (**B** and **D**) represent the mean value ± SD from each band calculated from four independent measurements. Significant differences between WT and FXN^I151F^ mice are indicated (p value < 0.01(**))

We also decided to analyze the content of protein-bound lipoic acid, a prosthetic group required for PDH activity. This analysis had two objectives: first, to complement the results from the targeted proteomics analysis, where several components of the PDH complex were analyzed; second, to further analyze the status of iron-sulfur clusters in FXN^I151F^ mice, as synthesis of this cofactor requires lipoate synthase, which is an iron-sulfur containing enzyme. Western blot analysis of cerebrum, cerebellum, and heart lysates using antibodies raised against lipoate revealed the presence of two bands, at 70 and 50 kDa apparent molecular weight (Figure 9). The 70 kDa band (which is more intense) corresponds to lipoic acid bound to DLAT (the E2 component of PDH), while three lipoic acid containing proteins migrate at 50 kDa. These are the E2 component of alpha-ketoglutarate dehydrogenase (Dihydrolipoyllysine succinyltransferase, DLST), the E2 component of the branched chain alpha-ketodehydrogenase complex, and the PDH-binding component X (a structural subunit of the PDH complex) [22]. As indicated in Figure 9, no significant differences were observed in DLAT-bound lipoic acid (70 kDa) between WT and FXN^I151F^ mice. Decreased content in the 50kDa band was observed in 21-week-old cerebrum, but not in the other tissues analyzed. This band was also decreased in 39-week-old mice, although here differences did not reach statistical significance. Overall, these results indicate that protein-bound lipoic acid biosynthesis (which requires an iron-sulfur enzyme) is not compromised in FXN^I151F^ mice.

## Discussion

In the present work we have analyzed the consequences of the FXN I151F mutation in mice, which is equivalent to the human I154F pathological mutation. This mutation was shown to cause partial loss of function [26, 27], decreased frataxin stability, and also accumulation of insoluble frataxin proteoforms [25]. This last observation suggested that these insoluble forms could be involved in the pathological mechanism of the I154F mutation. From our* in vivo* results, we can conclude that the primary biochemical consequence of this mutation is decreased frataxin content, as we have observed that FXN^I151F^ homozygous mice present very low frataxin levels in all tissues analyzed. In contrast, we dit not observe the presence of the insoluble frataxin proteoforms reported by Li and collaborators in cells overexpressing human FXN I154 [25]. As we have used similar protein extraction methods to those of the referenced work, we hypothesize that the presence of such insoluble intermediate forms *in vitro* was likely the consequence of frataxin overexpression under non-physiological conditions. Moreover, we observed that FXN^I151F^ mice present severe phenotypes which resemble those seen in other FA models. First, they present decreased weight gain, which is observed from 15 weeks onward. Second, they present neurological deficits, which manifest from week 23 onward. Third, they present marked biochemical alterations, which are observed before the appearance of the functional alterations (they are already seen in 21-week-old mice).

The behavioral tasks performed indicate that FXN^I151F^ mice exhibit several neurological deficits that are reminiscent of those observed in FA patients. Thus, FXN^I151F^ mice displayed decreased locomotor activity, defects in their forelimb muscular strength, reduced motor coordination and balance, and reduced hind and front limb stride length when compared with WT controls. Also, the results obtained indicate that neurological defects are progressive, as they are not manifested until 22 weeks of age. Indeed, most of them are observed at older ages. These defects may be caused by degeneration of dorsal root ganglia and of the dentate nucleus of the cerebellum, as observed in FA patients.

An interesting observation from this work is that biochemical alterations are observed before the onset of the functional disturbances, and that these alterations are not progressive in the nervous system (they are similar in 21- and 39-week-old mice). Among the proteins analyzed, we found decreased content of NDUFB8 (from complex I), SDHA and SDHB (from complex II), and ACO2. This observation is consistent with previous results in the KIKO mouse model, where complex I and II enzyme activities were also decreased in cerebella from asymptomatic mice [19]. Gene expression analysis indicates that loss of these proteins in cerebellum is caused by posttranscriptional events. As NDUF8, SDHA, SDHB, and ACO2 have in common that they either contain iron-sulfur clusters (SDHB and ACO2) or belong to complexes containing them (complex I and II), it could be hypothesized that these proteins are degraded due to the absence of this cofactor. Nevertheless, we should be cautious about this hypothesis, because FXN^I151F^ mice do not present a general loss of iron-sulfur clusters. This is evidenced by the high aconitase content and activity found in heart, and also by the normal presence of protein-bound lipoate under most of the conditions analyzed (protein-bound lipoate requires an iron-sulfur containing protein for its biosynthesis [35]). It is worth reminding that iron-sulfur loss is not a universal characteristic of frataxin deficiency [6, 36]. Therefore, the mechanism explaining the loss of these proteins in FXN^I151F^ mice may involve different regulatory pathways at either transcriptional or posttranscriptional levels. In this regard, ACO2, NDUF8, and SDHB are known to be regulated by posttranscriptional mechanisms. Studies in frataxin deficient yeasts indicate that aconitase and SDHB deficiency are caused by Cth2, an mRNA binding protein that regulates the expression of iron-containing proteins at the posttranscriptional level [37]. In mammals, ACO2, NDUF8, and SDHB are downregulated in conditions of iron deficiency [38], but the mechanisms causing this decrease are not the same for all of them. ACO2 downregulation is caused by the presence of an iron responsive element (IRE) in its mRNA, 5´ from the coding region. Upon iron starvation, Iron regulatory proteins (IRPs) bind to this site and block mRNA translation [39]. In contrast, NDUF8 and SDHB are not regulated by IRPs, as their mRNAs do not contain IREs. The existence of several regulatory mechanisms may explain why different mRNA and protein expression changes are observed in cerebellum and heart from FXN^I151F^ mice. In heart, changes in SDHB and NDUF8 content were observed both at the mRNA and protein level, while in cerebellum we observe decreased protein content with no changes in mRNA levels. These observations indicate that the mechanisms may be tissue specific and must include transcriptional and posttranscriptional events. Thus, a detailed focus on these events will be required to elucidate the mechanisms causing changes in the proteome of FXN^151F^ mice. Actually, IRP activation has been described in FA models [6], therefore, IRP activation could be one of these mechanisms.

The targeted proteomic analysis also revealed changes in SOD1 and SOD2 protein content. These alterations are more marked in heart than in the nervous system. Both enzymes are involved in superoxide scavenging and had been previously described as either induced or decreased in frataxin-deficient cells or mice [31, 40–42]. Interestingly, we observed both phenomena, as both enzymes are induced in 21-week-old mice, while their content is decreased in 39-week-old mice. The mechanism causing this paradoxical behavior could be complex, as several transcription factors are described to regulate superoxide dismutase expression. In this regard, the promoter of the *SOD1* gene contains binding sites for at least seven transcription factors, and at least two of these, NRF2 and NF-κB, could be responding to the oxidative stress conditions caused by frataxin deficiency [43]. Moreover, it has also been described that NRF2 signaling may be inhibited in frataxin-deficient cells [13]^14^. Thus, SOD expression would depend on the balance between activation of these transcription factors by stress, and inhibition of NRF2 signaling. Such balance could be different at different ages, explaining the differences observed between 21- and 39-week-old mice. It is worth commenting that a similar behavior was seen in hearts from the cardiac KO mouse model (MCK/frataxin), which showed a modest SOD2 induction between two and seven weeks followed by a dramatic decrease at later ages [7]. Another potential explanation for the SODs decline observed in 39-week-old hearts could be related to alterations in metal homeostasis caused by frataxin deficiency. In this regard, studies in yeast showed that the activities of both SOD1 and SOD2 were compromised due to limited availability of Cu and Mn, their metal cofactors. This limited Cu and Mn availability would be caused by interferences related to disrupted iron homeostasis [44].

In this work we also analyzed the content of three PDH containing components. We had previously observed decreased PDHA1 content and disturbances in the redox state of DLAT-bound lipoic acid in primary cultures of frataxin-deficient neonatal rat ventricular cardiomyocytes (NRVMs) [22]. These previous results indicated a 40% loss of PDHA1 content, and no major changes in DLAT or DLDH content. Indeed, treatment with the PDH activator dichloroacetate restored some of the defects found in frataxin-deficient NRVM [22]. The results obtained in the present work indicate a slight PDHA1 decrease in 21-week-old mice, while this protein was induced in hearts from 39-week-old mice. Regarding DLDH, we observed a 25 to 30% content loss in hearts and cerebellum from 39-week-old FXN^I151F^ mice. In this context, the previous results in rat cardiomyocytes indicated that DLAT-bound lipoic acid was more oxidized in frataxin-deficient cells, and the decreased DLDH content in FXN^I151F^ mice could cause a similar consequence. Therefore, we have observed alterations in the PDH complex, although the physiological consequences of these subtle differences, if any, would require further research.

Finally, the results from this work also indicate that the FXN^I151F^ mouse can be an excellent tool for analyzing the consequences of frataxin deficiency and for testing new therapies. Generating mouse models to recreate FA has been challenging, as this disease manifest when frataxin levels are below a pathological threshold. Therefore, animals must express some frataxin to be viable, but such expression may be lower than the pathological threshold required to trigger the disease. Actually, several FA mouse models have been generated, which can be classified into four main categories: 1) tissue specific KOs, which present a total loss of frataxin in some tissues; 2) GAA-repeat mice models, which harbor a GAA expansion in the frataxin gene; 3) an inducible mutant, in which frataxin expression is repressed by a shRNA transgene placed under the control of a TET promoter (doxycycline-inducible) [45]; 4) point mutation models, in which mouse frataxin has been genetically modified by CRISPR to present a pathological point mutation. All these models present limitations that could be overcome by the FXN^I151F^ model. Thus, tissue-specific KO mice [46] can be used to analyze the specific consequences of frataxin deficiency in a specific tissue, but they do not mimic the biochemical problem encountered by patients (the presence of limited amounts of frataxin in all tissues). GAA-repeat mice models have the advantage of mimicking the most common mutation found in patients (the GAA expansion). Nevertheless, all the GAA-repeat models developed to date present a mild phenotype, suggesting that the frataxin expression in these models is above (or slightly below) the pathological threshold required to trigger FA-like phenotypes in mice (reviewed in [47, 48]. This limits its usefulness as a model to study the pathophysiology of the disease and also to test new therapeutic approaches, as no clear biomarkers of disease severity can be defined. Regarding models based on point mutations, a partial characterization of a G127V FXN mouse model (equivalent to human pathological mutation G130V) was recently published. This model has a severe phenotype, as mice harboring this mutation presented a substantially reduced number of offspring. Mouse embryonic fibroblasts derived from this model exhibit significantly reduced proliferation and bioenergetic alterations [49], but there is no published data about the consequences of this mutation in adult mice. Therefore, the FXN^I151F^ mouse model is the only FA model which presents decreased frataxin content in all tissues and clear biomarkers. These features make this model an excellent tool for the study of FA pathophysiology and also for testing therapeutic approaches focused on frataxin replacement (i.e., gene therapy) or on preventing the consequences of frataxin deficiency. On the other hand, it is not suitable for testing several therapeutic candidates, like those drugs focused on increasing endogenous frataxin expression.

In summary, the results obtained indicate that the pathological mechanisms of the FXN I154F mutation are triggered by decreased frataxin content as i) decreased content of frataxin is found in all tissues analyzed, ii) the presence of insoluble intermediate forms was not observed, and iii) the phenotypes observed resemble those seen in FA patients and models. These observations are consistent with clinical data, as the clinical phenotype of patients carrying the I154F mutation (compound heterozygotes for the GAA triplet-repeat expansion and the point mutation) is similar to that of individuals homozygous for the GAA expansion [24]. Therefore, patients carrying this mutation would benefit from gene therapy or other frataxin replacement therapies. Our results also indicate that the FXN^I151F^ mouse is an excellent model for FA research, which can be used for investigating the pathological consequences of frataxin deficiency. In this regard, the presence of clear biochemical and functional biomarkers indicates that this model can be used to test the effect of several therapeutic approaches.

## Supplementary Information

Below is the link to the electronic supplementary material.

Supplementary file1 (XLSX 13 KB)

Supplementary file2 (PDF 561 KB)

Supplementary file3 (PDF 484 KB)

## Data Availability

Mass spectrometry data produced in this study is available in Panorama Public. https://panoramaweb.org/FxnI151FMs.url
