# Supplementary material for: Mice harboring the FXN I151F pathological point mutation present decreased frataxin levels, a Friedreich ataxia-like phenotype, and mitochondrial alterations
Source: Cell Mol Life Sci. 2022 Jan 17;79(2):74. doi: 10.1007/s00018-021-04100-5 (PMC8763788; doi:10.1007/s00018-021-04100-5)
Supplement: Supplementary file 2 — Supplementary file2 (PDF 561 KB) [file 18_2021_4100_MOESM2_ESM.pdf]

Supplemental Table 2, Transitions used in the MRM analysis

Protein Uniprot Code	Peptide Sequence	Precursor Mz	Isotope	Precursor Charge	Product Mz	Product Charge	Fragment Ion
Q9CQA3	DLVPDLSNFYAQYK	836,91	light	2	1345,64	1	y11
		836,91	light	2	1020,48	1	y8
		836,91	light	2	673,32	2	y11
		840,92	heavy	2	1353,66	1	y11
		840,92	heavy	2	1028,49	1	y8
		840,92	heavy	2	677,33	2	y11
Q9CQA3	LQDPFSVYR	562,79	light	2	357,18	1	b3
		562,79	light	2	883,43	1	y7
		562,79	light	2	768,40	1	y6
		567,79	heavy	2	357,18	1	b3
		567,79	heavy	2	893,44	1	y7
		567,79	heavy	2	778,41	1	y6
P21550	GNPTVEVDLHTAK	690,86	light	2	912,48	1	y8
		690,86	light	2	783,44	1	y7
		690,86	light	2	605,33	2	y11
		694,87	heavy	2	920,49	1	y8
		694,87	heavy	2	791,45	1	y7
		694,87	heavy	2	609,33	2	y11
P17182	IGAEVYHNLK	381,88	light	3	674,36	1	y5
		381,88	light	3	511,30	1	y4
		381,88	light	3	515,77	2	y9
		384,55	heavy	3	682,38	1	y5
		384,55	heavy	3	519,31	1	y4
		384,55	heavy	3	519,78	2	y9
P17182	DATNVGDEGGFAPNILENK	654,31	light	3	898,50	1	y8
		654,31	light	3	827,46	1	y7
		654,31	light	3	414,23	2	y7
		656,98	heavy	3	906,51	1	y8
		656,98	heavy	3	835,48	1	y7
		656,98	heavy	3	418,24	2	y7

P62897	ADLIAYLK	453,77	light	2	494,30	1	y4
		453,77	light	2	423,26	1	y3
		453,77	light	2	260,20	1	y2
		457,78	heavy	2	502,31	1	y4
		457,78	heavy	2	431,27	1	y3
		457,78	heavy	2	268,21	1	y2
P62897	TGPNLHGLFGR	390,21	light	3	534,29	2	y10
		390,21	light	3	505,78	2	y9
		390,21	light	3	337,52	3	y9
		393,55	heavy	3	539,30	2	y10
		393,55	heavy	3	510,78	2	y9
		393,55	heavy	3	340,86	3	y9
Q9D0M3	AANNGALPPDLSYIVR	835,95	light	2	1172,67	1	y10
		835,95	light	2	1059,58	1	y9
		835,95	light	2	530,30	2	y9
		840,95	heavy	2	1182,68	1	y10
		840,95	heavy	2	1069,59	1	y9
		840,95	heavy	2	535,30	2	y9
Q9D0M3	LSDYFPKPYPNPEAAR	622,31	light	3	876,42	2	y15
		622,31	light	3	832,91	2	y14
		622,31	light	3	693,86	2	y12
		625,65	heavy	3	881,43	2	y15
		625,65	heavy	3	837,91	2	y14
		625,65	heavy	3	698,87	2	y12
Q9D0M3	GLLSSLDHTSIR	433,57	light	3	564,80	2	y10
		433,57	light	3	508,26	2	y9
		433,57	light	3	464,75	2	y8
		436,91	heavy	3	569,81	2	y10
		436,91	heavy	3	513,27	2	y9
		436,91	heavy	3	469,75	2	y8
Q03265	ILGADTSVDLEETGR	788,40	light	2	1349,62	1	y13
		788,40	light	2	1005,48	1	y9
		788,40	light	2	819,38	1	y7

		793,40	heavy	2	1359,63	1	y13
		793,40	heavy	2	1015,49	1	y9
		793,40	heavy	2	829,39	1	y7
Q03265	HALIIYDDLSK	644,35	light	2	209,10	1	b2
		644,35	light	2	1079,60	1	y9
		644,35	light	2	853,43	1	y7
		648,36	heavy	2	209,10	1	b2
		648,36	heavy	2	1087,61	1	y9
		648,36	heavy	2	861,44	1	y7
P56480	LVLEVAQHLGESTVR	550,98	light	3	213,16	1	b2
		550,98	light	3	719,89	2	y13
		550,98	light	3	663,34	2	y12
		554,31	heavy	3	213,16	1	b2
		554,31	heavy	3	724,89	2	y13
		554,31	heavy	3	668,35	2	y12
P56480	AIAELGIYPAVDPLDSTSR	663,35	light	3	775,39	1	y7
		663,35	light	3	579,29	2	y11
		663,35	light	3	388,20	2	y7
		666,69	heavy	3	785,40	1	y7
		666,69	heavy	3	584,30	2	y11
		666,69	heavy	3	393,21	2	y7
P00405	LLEVDNR	429,74	light	2	745,38	1	y6
		429,74	light	2	632,30	1	y5
		429,74	light	2	503,26	1	y4
		434,74	heavy	2	755,39	1	y6
		434,74	heavy	2	642,31	1	y5
		434,74	heavy	2	513,27	1	y4
P00405	VVLPMELPIR	583,85	light	2	855,48	1	y7
		583,85	light	2	484,78	2	y8
		583,85	light	2	428,24	2	y7
		588,86	heavy	2	865,48	1	y7
		588,86	heavy	2	489,79	2	y8
		588,86	heavy	2	433,25	2	y7

P00405	MLISSEDVLHSWAVPSLGLK	728,06	light	3	969,52	2	y18
		728,06	light	3	912,98	2	y17
		730,73	heavy	3	973,53	2	y18
		730,73	heavy	3	916,99	2	y17
Q9DB77	LPNGLVIASLENYAPLSR	643,03	light	3	692,88	2	b13
		643,03	light	3	543,32	1	y5
		643,03	light	3	472,29	1	y4
		646,36	heavy	3	692,88	2	b13
		646,36	heavy	3	553,33	1	y5
		646,36	heavy	3	482,30	1	y4
Q9DB77	AVAQGNLSSADVQAAK	765,40	light	2	1160,59	1	y12
		765,40	light	2	876,44	1	y9
		765,40	light	2	644,83	2	y13
		769,41	heavy	2	1168,60	1	y12
		769,41	heavy	2	884,46	1	y9
		769,41	heavy	2	648,84	2	y13
Q9DB77	NALANPLYC[+57]PDYR	783,87	light	2	299,17	1	b3
		783,87	light	2	1083,49	1	y8
		783,87	light	2	710,29	1	y5
		788,88	heavy	2	299,17	1	b3
		788,88	heavy	2	1093,50	1	y8
		788,88	heavy	2	720,30	1	y5
Q8K2B3	VTLEYRPVIDK	444,92	light	3	617,35	2	y10
		444,92	light	3	566,82	2	y9
		444,92	light	3	510,28	2	y8
		447,59	heavy	3	621,35	2	y10
		447,59	heavy	3	570,83	2	y9
		447,59	heavy	3	514,29	2	y8
Q8K2B3	LGANSLLDLVVFGR	737,42	light	2	918,54	1	y8
		737,42	light	2	805,46	1	y7
		737,42	light	2	690,43	1	y6
		742,43	heavy	2	928,55	1	y8
		742,43	heavy	2	815,46	1	y7

		742,43	heavy	2	700,44	1	y6
P08228	VISLSGEHSIIGR	684,39	light	2	1155,61	1	y11
		684,39	light	2	955,50	1	y9
		684,39	light	2	578,31	2	y11
		689,39	heavy	2	1165,62	1	y11
		689,39	heavy	2	965,50	1	y9
		689,39	heavy	2	583,31	2	y11
P08228	DGVANVSIEDR	587,79	light	2	903,45	1	y8
		587,79	light	2	832,42	1	y7
		587,79	light	2	619,30	1	y5
		592,79	heavy	2	913,46	1	y8
		592,79	heavy	2	842,42	1	y7
		592,79	heavy	2	629,31	1	y5
P09671	AIWNVINWENVTER	872,44	light	2	584,32	1	b5
		872,44	light	2	1160,57	1	y9
		872,44	light	2	1047,49	1	y8
		877,45	heavy	2	584,32	1	b5
		877,45	heavy	2	1170,58	1	y9
		877,45	heavy	2	1057,49	1	y8
P09671	GDVTTQVALQPALK	720,91	light	2	1013,53	1	b10
		720,91	light	2	556,35	1	y5
		720,91	light	2	428,29	1	y4
		724,91	heavy	2	1013,53	1	b10
		724,91	heavy	2	564,36	1	y5
		724,91	heavy	2	436,30	1	y4
O08749	ALTGGIAHLFK	564,33	light	2	943,54	1	y9
		564,33	light	2	842,49	1	y8
		564,33	light	2	472,27	2	y9
		568,34	heavy	2	951,55	1	y9
		568,34	heavy	2	850,50	1	y8
		568,34	heavy	2	476,28	2	y9
O08749	RPFTQNLGLEELGIELDPK	723,72	light	3	849,45	2	b15
		723,72	light	3	771,42	1	y7

		726,39	heavy	3	849,45	2	b15
		726,39	heavy	3	779,44	1	y7
Q8BMF4	VAPAPAGVFTDIPISNIR	919,51	light	2	699,41	1	y6
		919,51	light	2	834,46	2	y16
		919,51	light	2	750,41	2	y14
		924,52	heavy	2	709,42	1	y6
		924,52	heavy	2	839,46	2	y16
		924,52	heavy	2	755,42	2	y14
Q8BMF4	GLETIASDVVSLASK	745,41	light	2	976,53	1	y10
		745,41	light	2	905,49	1	y9
		745,41	light	2	505,30	1	y5
		749,42	heavy	2	984,55	1	y10
		749,42	heavy	2	913,51	1	y9
		749,42	heavy	2	513,31	1	y5
P35486	LEEGPPVTTVLTR	706,39	light	2	1040,61	1	y10
		706,39	light	2	983,59	1	y9
		706,39	light	2	492,30	2	y9
		711,40	heavy	2	1050,62	1	y10
		711,40	heavy	2	993,60	1	y9
		711,40	heavy	2	497,30	2	y9
P35486	AILAELTGR	472,28	light	2	759,44	1	y7
		472,28	light	2	646,35	1	y6
		472,28	light	2	575,31	1	y5
		477,29	heavy	2	769,44	1	y7
		477,29	heavy	2	656,36	1	y6
		477,29	heavy	2	585,32	1	y5
Q9CZU6	GLVYETSVLDPDEGIR	881,95	light	2	914,46	1	y8
		881,95	light	2	801,37	1	y7
		881,95	light	2	686,35	1	y6
		886,95	heavy	2	924,47	1	y8
		886,95	heavy	2	811,38	1	y7
		886,95	heavy	2	696,36	1	y6
Q9CZU6	DYIWNTLNSGR	669,83	light	2	279,10	1	b2

		669,83	light	2	947,47	1	y8
		669,83	light	2	761,39	1	y7
		674,83	heavy	2	279,10	1	b2
		674,83	heavy	2	957,48	1	y8
		674,83	heavy	2	771,40	1	y7
P52480	IYVDDGLISLQVK	731,91	light	2	277,15	1	b2
		731,91	light	2	1186,67	1	y11
		731,91	light	2	574,36	1	y5
		735,92	heavy	2	277,15	1	b2
		735,92	heavy	2	1194,68	1	y11
		735,92	heavy	2	582,37	1	y5
P52480	GADFLVTEVENGGSLGSK	890,44	light	2	504,25	1	b5
		890,44	light	2	1177,57	1	y12
		890,44	light	2	848,41	1	y9
		894,45	heavy	2	504,25	1	b5
		894,45	heavy	2	1185,58	1	y12
		894,45	heavy	2	856,43	1	y9
P52480	GDLGIEIPAEK	571,31	light	2	856,48	1	y8
		571,31	light	2	686,37	1	y6
		571,31	light	2	444,25	1	y4
		575,32	heavy	2	864,49	1	y8
		575,32	heavy	2	694,39	1	y6
		575,32	heavy	2	452,26	1	y4
		625,34	light	2	794,44	1	y7
		629,35	heavy	2	802,45	1	y7
Q99KI0	NAVTQEFGPVPDTAR	801,40	light	2	959,49	1	y9
		801,40	light	2	755,40	1	y7
		801,40	light	2	559,28	1	y5
		806,40	heavy	2	969,50	1	y9
		806,40	heavy	2	765,41	1	y7
		806,40	heavy	2	569,29	1	y5
Q99KI0	DLEDLQILIK	600,35	light	2	842,53	1	y7
		600,35	light	2	614,42	1	y5

		604,35	heavy	2	850,55	1	y7
		604,35	heavy	2	622,44	1	y5
P16858	GAAQNIIPASTGAAK	685,38	light	2	555,29	1	b6
		685,38	light	2	815,46	1	y9
		685,38	light	2	702,38	1	y8
		689,38	heavy	2	555,29	1	b6
		689,38	heavy	2	823,48	1	y9
		689,38	heavy	2	710,39	1	y8
P16858	LVINGKPITIFQER	543,32	light	3	708,40	2	y12
		543,32	light	3	651,86	2	y11
		543,32	light	3	472,61	3	y12
		546,66	heavy	3	713,41	2	y12
		546,66	heavy	3	656,87	2	y11
		546,66	heavy	3	475,94	3	y12
Actins (housekeeping)	SYELPDGQVITIGNER	895,95	light	2	1086,59	1	y10
		895,95	light	2	689,36	1	y6
		895,95	light	2	475,23	1	y4
		900,95	heavy	2	1096,60	1	y10
		900,95	heavy	2	699,37	1	y6
		900,95	heavy	2	485,23	1	y4
Actins (housekeeping)	AGFAGDDAPR	488,73	light	2	701,32	1	y7
		488,73	light	2	630,28	1	y6
		488,73	light	2	573,26	1	y5
		493,73	heavy	2	711,33	1	y7
		493,73	heavy	2	640,29	1	y6
		493,73	heavy	2	583,27	1	y5
P38647	VQQTVQDLFGR	645,84	light	2	1063,55	1	y9
		645,84	light	2	935,49	1	y8
		645,84	light	2	735,38	1	y6
		650,85	heavy	2	1073,56	1	y9
		650,85	heavy	2	945,50	1	y8
		650,85	heavy	2	745,39	1	y6
P38647	LLGQFTLIGIPPAPR	796,98	light	2	820,50	1	y8

		796,98	light	2	707,42	1	y7
		796,98	light	2	537,31	1	y5
		801,98	heavy	2	830,51	1	y8
		801,98	heavy	2	717,43	1	y7
		801,98	heavy	2	547,32	1	y5
P63038	AAVEEGIVLGGGC[+57]ALLR	842,96	light	2	1015,57	1	y10
		842,96	light	2	916,50	1	y9
		842,96	light	2	803,42	1	y8
		847,96	heavy	2	1025,58	1	y10
		847,96	heavy	2	926,51	1	y9
		847,96	heavy	2	813,43	1	y8
P17183	IAPALISSGISVVEQEK	870,99	light	2	1162,60	1	y11
		870,99	light	2	818,43	1	y7
		870,99	light	2	778,93	2	y15
		875,00	heavy	2	1170,61	1	y11
		875,00	heavy	2	826,44	1	y7
		875,00	heavy	2	782,94	2	y15
P17183	YITGDQLGALYQDFVR	929,97	light	2	1181,63	1	y10
		929,97	light	2	1068,55	1	y9
		929,97	light	2	827,40	1	y6
		934,97	heavy	2	1191,64	1	y10
		934,97	heavy	2	1078,56	1	y9
		934,97	heavy	2	837,41	1	y6
