# Supplementary material for: Mice harboring the FXN I151F pathological point mutation present decreased frataxin levels, a Friedreich ataxia-like phenotype, and mitochondrial alterations
Source: Cell Mol Life Sci. 2022 Jan 17;79(2):74. doi: 10.1007/s00018-021-04100-5 (PMC8763788; doi:10.1007/s00018-021-04100-5)

Supplemental figure 1

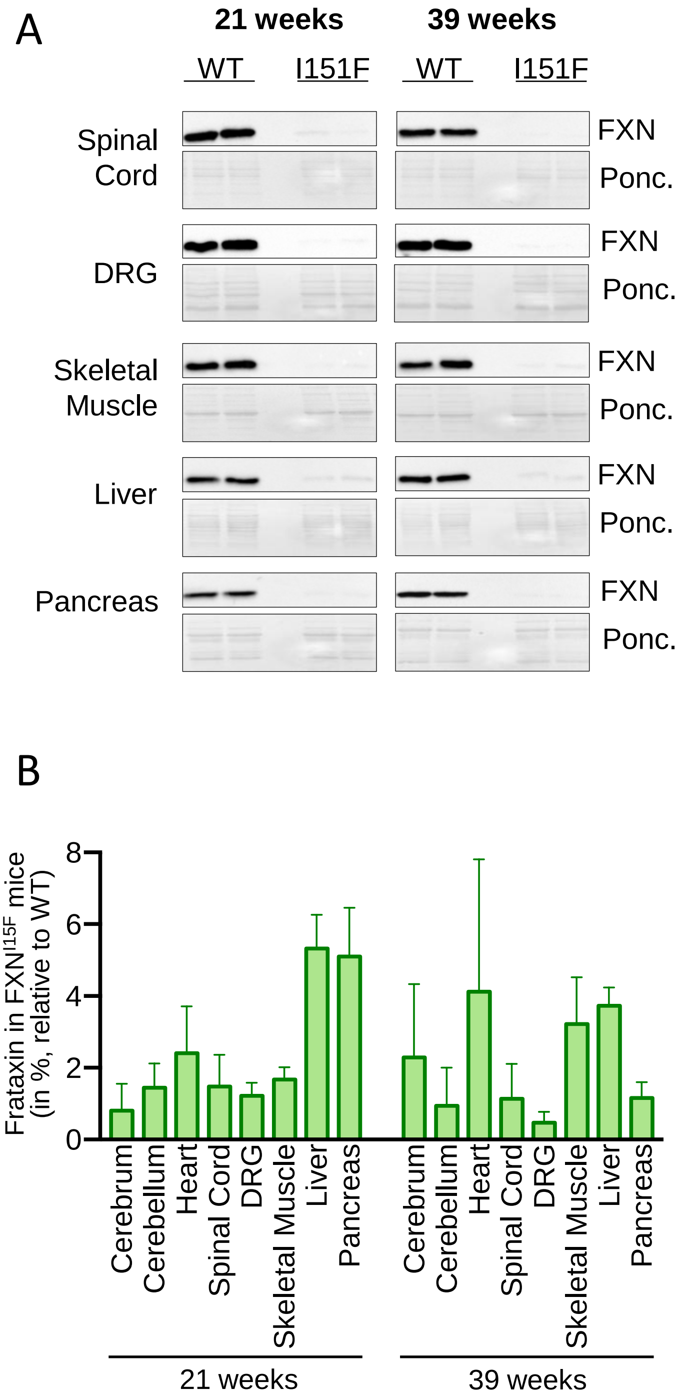

Supplemental figure 2

A

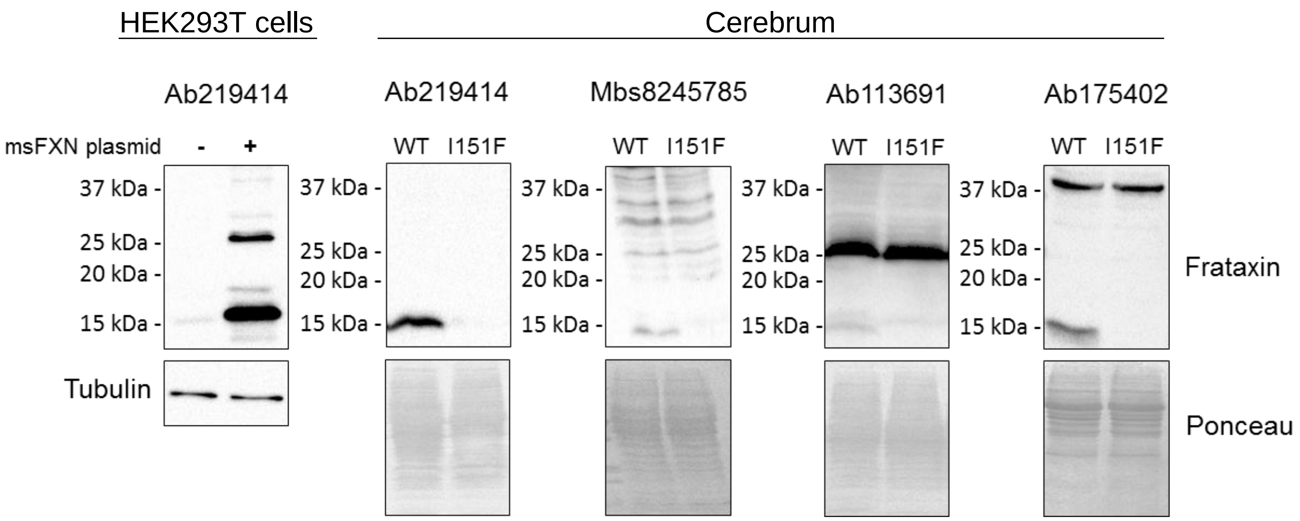

B

Peptide Sequence	*ESS*	*Precursor Charge*	*Product Ions*	*Detected*
NWVYSHDGVSLHELLAR^{185}	0.92	2+	y8+, y10+, y11+	No
LDLSSLAYSGK^{205}	0.68	2+	b2+, y8+, y9+	No
LGGDLGTYVINK^{144}	0.66	2+	y7+, y8+, y9+	Yes
QIWLSSPSSGPK^{161}	0.63	2+	y7+, y8+, y10+	No

C

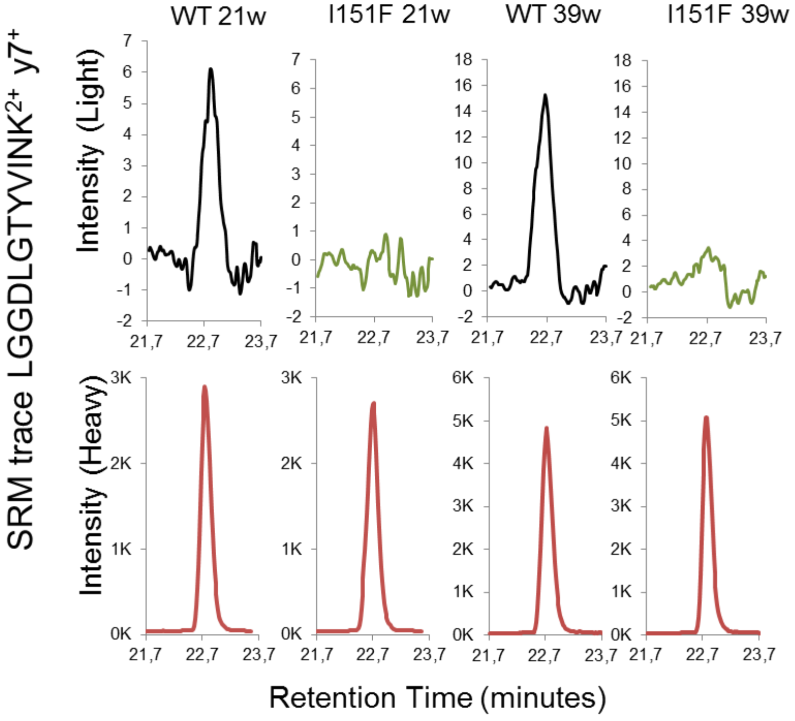

Supplemental figure 3

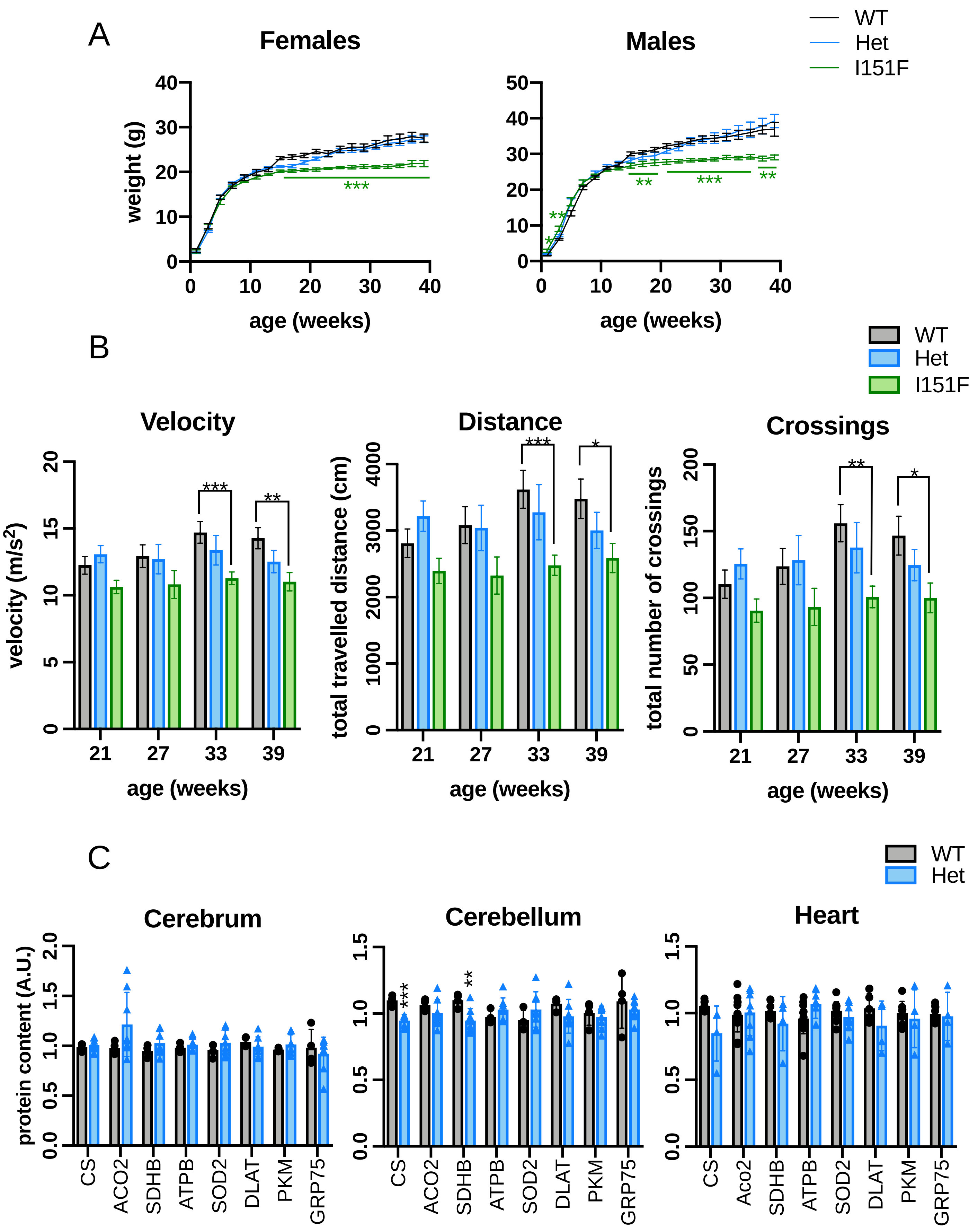

Supplemental figure 4

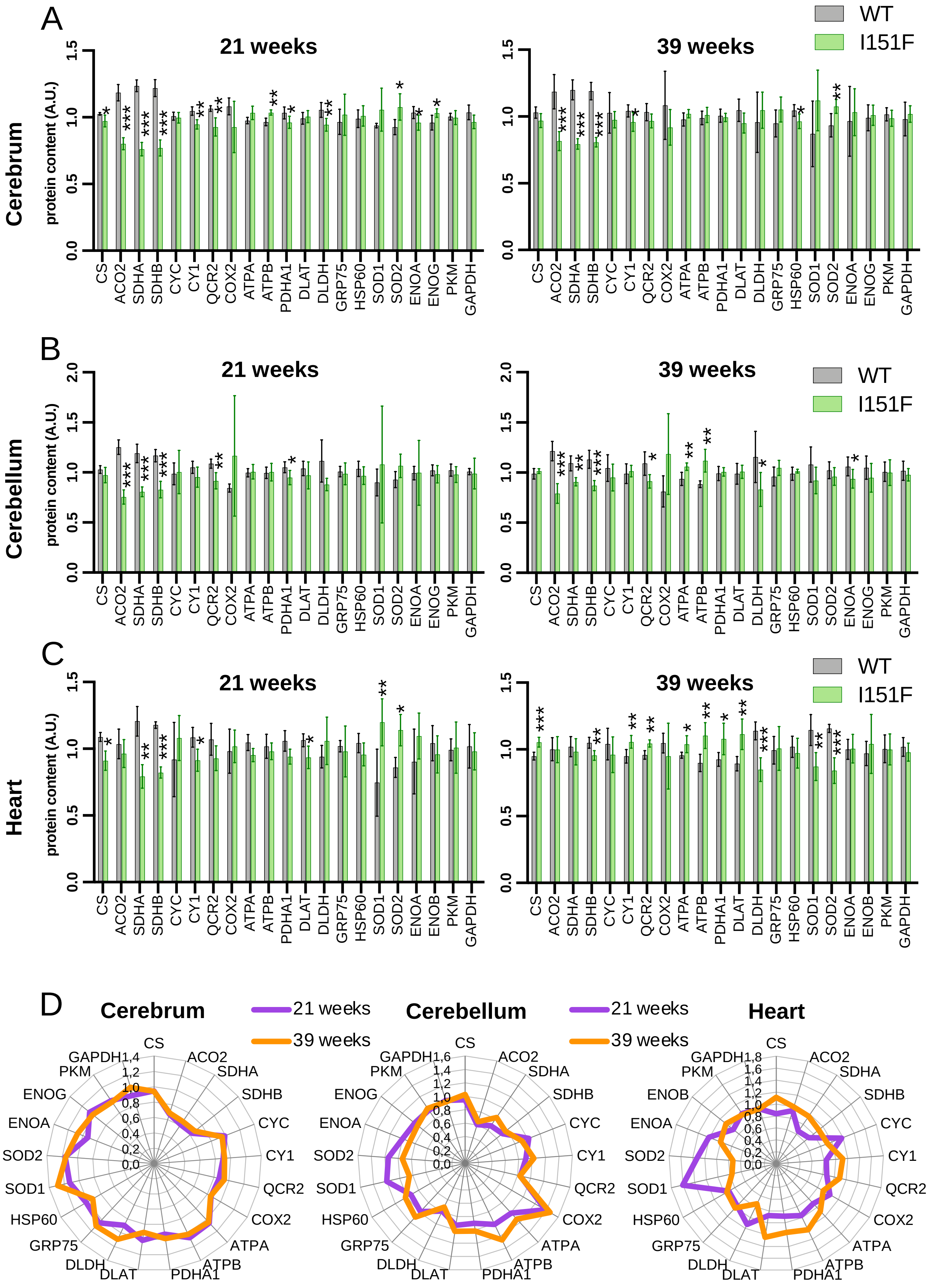

Supplemental figure 5

A

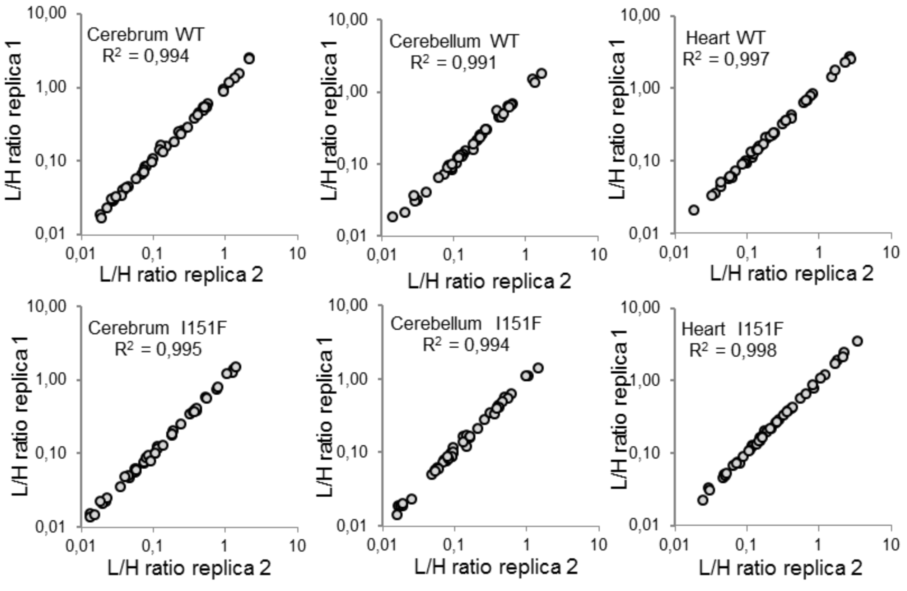

Mean R^2 correlation value technical replicates

**Tissue**	**21 weeks**	**39 weeks**
Cerebrum	0.988	0.996
Cerebellum	0.986	0.994
Heart	0.996	0.988

B

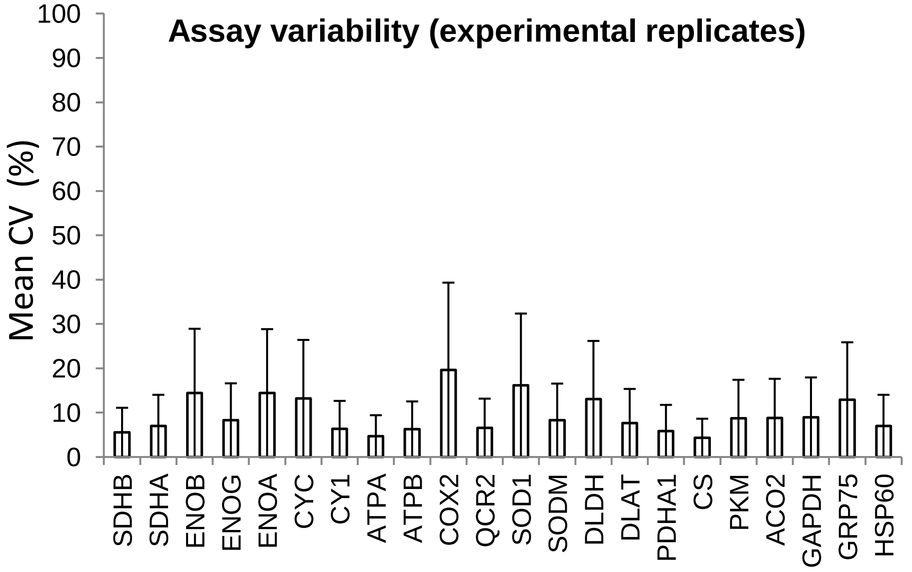

Supplement: Supplementary file 3 — Supplementary file3 (PDF 484 KB) [file 18_2021_4100_MOESM3_ESM.pdf]
